# A novel subtype of sporadic Creutzfeldt–Jakob disease with *PRNP* codon 129MM genotype and PrP plaques

**DOI:** 10.1007/s00401-023-02581-1

**Published:** 2023-05-08

**Authors:** Rabeah Bayazid, Christina Orru’, Rabail Aslam, Yvonne Cohen, Amelia Silva-Rohwer, Seong-Ki Lee, Rossana Occhipinti, Qingzhong Kong, Shashirekha Shetty, Mark L. Cohen, Byron Caughey, Lawrence B. Schonberger, Brian S. Appleby, Ignazio Cali

**Affiliations:** 1 Department of Pathology, School of Medicine, Case Western Reserve University, Cleveland, OH USA; 2 Department of Physiology and Biophysics, School of Medicine, Case Western Reserve University, Cleveland, OH USA; 3 Department of Neurology, School of Medicine, Case Western Reserve University, Cleveland, OH USA; 4 Department of Psychiatry, School of Medicine, Case Western Reserve University, Cleveland, OH USA; 5 National Prion Disease Pathology Surveillance Center, Cleveland, OH USA; 6 Laboratory of Persistent Viral Diseases, NIH, Hamilton, MT USA; 7 Division of High-Consequence Pathogens and Pathology, National Center for Emerging and Zoonotic Infectious Diseases, Centers for Disease Control and Prevention, Atlanta, GA USA

**Keywords:** Prion disease, PrP plaques, White matter, Gray matter, Prion strain, Phenotype

## Abstract

The presence of amyloid kuru plaques is a pathological hallmark of sporadic Creutzfeldt–Jakob disease (sCJD) of the MV2K subtype. Recently, PrP plaques (p) have been described in the white matter of a small group of CJD (p-CJD) cases with the 129MM genotype and carrying resPrP^D^ type 1 (T1). Despite the different histopathological phenotype, the gel mobility and molecular features of p-CJD resPrP^D^ T1 mimic those of sCJDMM1, the most common human prion disease. Here, we describe the clinical features, histopathology, and molecular properties of two distinct PrP plaque phenotypes affecting the gray matter (p^GM^) or the white matter (p^WM^) of sCJD cases with the PrP 129MM genotype (sCJDMM). Prevalence of p^GM^- and p^WM^-CJD proved comparable and was estimated to be ~ 0.6% among sporadic prion diseases and ~ 1.1% among the sCJDMM group. Mean age at onset (61 and 68 years) and disease duration (~ 7 months) of p^WM^- and p^GM^-CJD did not differ significantly. PrP plaques were mostly confined to the cerebellar cortex in p^GM^-CJD, but were ubiquitous in p^WM^-CJD. Typing of resPrP^D^ T1 showed an unglycosylated fragment of ~ 20 kDa (T1^20^) in p^GM^-CJD and sCJDMM1 patients, while a doublet of ~ 21–20 kDa (T1^21−20^) was a molecular signature of p^WM^-CJD in subcortical regions. In addition, conformational characteristics of p^WM^-CJD resPrP^D^ T1 differed from those of p^GM^-CJD and sCJDMM1. Inoculation of p^WM^-CJD and sCJDMM1 brain extracts to transgenic mice expressing human PrP reproduced the histotype with PrP plaques only in mice challenged with p^WM^-CJD. Furthermore, T1^20^ of p^WM^-CJD, but not T1^21^, was propagated in mice. These data suggest that T1^21^ and T1^20^ of p^WM^-CJD, and T1^20^ of sCJDMM1 are distinct prion strains. Further studies are required to shed light on the etiology of p-CJD cases, particularly those of T1^20^ of the novel p^GM^-CJD subtype.

**Supplementary Information:**

The online version contains supplementary material available at 10.1007/s00401-023-02581-1.

## Introduction

Human prion diseases are a group of rare, invariably fatal, rapidly progressive, neurodegenerative disorders characterized by the misfolding of the cellular prion protein (PrP^C^) into the disease-associated, pathogenic form (PrP^D^) [24]. The PrP^C^ to PrP^D^ structural conversion is accompanied by an increase in β-sheets motifs at the expense of α-helical domains, leading to an increased resistance to enzymatic degradation by proteases [3, 15, 41]. The result of these pathogenic events is the accumulation of different species of PrP^D^ aggregates in the central nervous system, giving rise to several clinico-histopathological phenotypes commonly associated with distinct prion strains (i.e., conformers of PrP^D^) [22, 62]. Unlike other neurodegenerative disorders, in addition to idiopathic events or mutations to the PrP gene (*PRNP*), human prion diseases can also be acquired by infection (i.e., iatrogenic etiology) [5]. While the idiopathic and genetic forms account for approximately 85% and 15%, respectively, the iatrogenic form is rare (< 1%) [5].

Sporadic Creutzfeldt–Jakob disease (sCJD), the most common human prion disease, has been classified into 6 groups (consolidated into 5 subtypes) based on the pairing of two major molecular determinants of the disease phenotype. The first molecular determinant is the genotype at codon 129 of the *PRNP*, which encodes for methionine (M) or valine (V), allowing for three genetic variants: the homozygous 129MM and 129VV genotypes, or the heterozygous 129MV genotype. The second molecular determinant is the resPrP^D^, the C-terminal portion of PrP^D^ that is resistant to proteolytic digestion; cases are classified as either type 1 or type 2 according to the molecular mass of the unglycosylated isoform of resPrP^D^ [48]. While the size of resPrP^D^ type 1 (T1) can be ~ 21 kDa or ~ 20 kDa depending on the buffer pH, the molecular mass of the unglycosylated resPrP^D^ type 2 (T2) isoform is ~ 19 kDa independent of buffer pH [50]. Therefore, the five sCJD subtypes are MM(MV)1, VV1, MM2, MV2 and VV2 [55, 56].

Recent findings have highlighted that different histopathological phenotypes (histotypes) of sCJDMV2 are the result of a differential expression of PrP^C^-129 M and -129 V alleles, which, in turn, are converted into PrP^D^-129 M and PrP^D^-129 V [25, 49]. To further add to the phenotypic heterogeneity, mixed PrP^D^ types are observed in approximately 40–60% of sCJD cases [8, 14].

Histopathologically, sCJDMV2K, is the only sCJD histotype characterized by the presence of PrP plaques of the kuru-type [62]. However, PrP plaques have recently been described in the white matter of a small group of CJD cases (p-CJD) with codon 129MM genotype and resPrP^D^ T1 in most cases (p-CJDMM1) [4, 26, 33, 63]. These studies indicated that resPrP^D^ T1 molecular features of p-CJDMM1 and sCJDMM1 were similar [26, 33, 63]. Furthermore, bank voles inoculated with brain extracts of p-CJDMM1 failed to reproduce the PrP plaque histotype, and instead showed common sCJDMM1 pathological features [63]. In two other independent studies, PrP plaques were shown to affect the cerebral cortex in one patient and only the cerebellar cortex in another [31, 64]. Both cases had the 129MM genotype, and were later reported to likely be iatrogenic [35]. This conclusion was proposed based on laboratory findings given that one of the patient was a neurosurgeon, but had no recognized iatrogenic risk factors [64]. The second patient underwent brain surgery without a dura mater (DM) graft [31]. Moreover, in vivo experiments with brain suspensions of these two cases revealed histotypes and PrP molecular features similar to those of a subset of DM-associated iatrogenic CJD (DM-iCJD) featuring PrP plaques [35]. While several studies have shown the presence of PrP plaques in iCJD-129MM patients linked to DM or prion-contaminated cadaveric growth hormone (GH) [13, 34, 61], it remains unclear whether gel mobility of the resPrP^D^, often identified as “type intermediate" or “type i”, is ubiquitous in all iCJD-129MM cases [20, 66].

Beyond sporadic CJD, PrP plaques, including florid plaques and other less common types of plaques, have been described in iCJD, kuru, and variant CJD in humans, or in bovine spongiform encephalopathy (BSE) and chronic wasting disease (CWD) in animals [13, 16, 30, 43, 72]. These prion diseases are readily transmissible to certain transgenic mice and to humans [17, 19, 34, 69]. Furthermore, there are concerns about whether CWD prions can infect humans, as humans are exposed to CWD in several states [29]. Plaques are also observed in genetic prion diseases, such as Gerstmann-Sträussler-Scheinker syndrome (GSS) [18].

In the present study, we retrospectively examined the histopathology of 620 sCJD cases carrying the 129MM genotype that were referred to the National Prion Disease Pathology Surveillance Center (NPDPSC) in the United States between 2012 and 2017. From this assessment we found PrP plaques in the brain of 14 cases (2.2%). Two groups of 7 cases each harbored PrP in the white matter (p^WM^-CJD) throughout the brain, or in the gray matter (p^GM^-sCJD), typically in the cerebellar cortex. Seven additional p-CJD cases, found independently of the retrospective examination, brought the total number of p-CJD cases to 21. Here, we describe the detailed disease phenotype and distinctive molecular features of PrP^D^ associated with the p^WM^-CJD group and novel p^GM^-CJD subtype. These findings are important as they point toward the distinction of two human prion diseases by divergent PrP plaque phenotypes, and highlight the importance of a careful histopathological examination, with special attention to the cerebellar cortex.

## Materials and methods

### Case series

Autopsied brains from 620 sCJD cases with the homozygous methionine (M) genotype at codon 129 of the PrP gene (sCJDMM) were referred to the NPDPSC between 2012 and 2017. PrP^D^ typing was carried out on routine western blot examination of 3 brain regions: frontal cortex, occipital cortex, and cerebellum. 75% (*n* = 463) of the 620 sCJD cases showed resPrP^D^ T1 and were diagnosed as sCJDMM1. 8% (*n* = 48) harbored resPrP^D^ T2, and 17% (*n* = 109) had a co-occurrence of resPrP^D^ types 1 and 2 (T1-2); they were diagnosed as sCJDMM2 and -MM1-2, respectively. These sCJD cases underwent a retrospective histopathological re-characterization of the cerebellum for the identification of PrP plaques on hematoxylin–eosin (H&E) and PrP immunostained sections. Seven additional sCJDMM cases harboring PrP plaques (cases 9–12, 14, 16, 19, Table 1), identified before (*n* = 5) or after (*n* = 2) the retrospective examination, were included in the study. Well-characterized sCJDMM1 and -MM2 cases were used as controls for further molecular studies [8, 9].

**Table 1:** Case-cohort of US CJD cases with PrP plaques (p) affecting the gray matter (p^GM^-CJD) and white matter (p^WM^-CJD)

Case number		PK-resistant PrP^D^			Gender, female (%)	Race, white (%)	Age at onset (years)	Disease duration (months)	Cases found by retrospective study ^b^	Available frozen tissue subcortical regions
	Year case ID	resPrP^D^ Type	T2^a^ (%)							
			Brain	Crbl						
p^GM^-CJD										
1	2015	1–2	98	ND	–	W	63	12	Yes	Yes
2	2016	1–2	89	100	+	W	73	4	Yes	Yes
3	2015	1–2	45	10	–	W	68	24	Yes	Yes
4	2016	1–2	29	0	+	W	71	2	Yes	Yes
5	2015	1–2	26	0	–	W	57	2	Yes	Yes
6	2016	1–2	16	0	+	W	60	2	Yes	Yes
7	2014	1–2	3	0	+	W	83	2	Yes	Yes
			44 ± 36^e^	18 ± 40^e^	57	100	67 ± 9^e^	7 ± 8^e^	100%	100%
p^WM^-CJD										
8	2017	1–2	60	ND	+	W	72	9	Yes	Yes
9^c^	2005	1–2	50	0	–	W	54	11	No	Yes
10^d^	2018	1–2	40	0	–	A	69	7	No	Yes
11^c^	2003	1–2	24	0	–	W	52	11	No	Yes
12^c^	2006	1–2	8	0	–	W	59	7	No	Yes
13	2015	1–2	5	ND	–	W	75	1	Yes	Yes
14^d^	2019	1–2	5	0	–	W	51	7	No	Yes
15	2016	1–2	4	0	+	W	72	9	Yes	Yes
16^c^	2010	1–2	2	0	–	W	57	13	No	Yes
17	2015	1	0	0	–	W	62	7	Yes	Yes
18	2016	1	0	0	–	W	44	3	Yes	Yes
19^c^	2011	1	0	0	–	U	76	5	No	No
20	2013	1	0	NA	–	W	68	2	Yes	No
21	2016	1	0	0	+	W	50	11	Yes	No
			14 ± 21^e,f^	0	21	92	61.5 ± 10.5^e^	7 ± 4^e^	50%	79%

*ID* identification, *ND* not detected, *NA* not available, *M* male, *F* female, *W* white, *A* Asian, *U* unknown

^a^Percentage of PK-resistant PrP^D^ (resPrP^D^) T2 out of total resPrP^D^ (total resPrP^D^ = resPrP^D^ T1 + resPrP^D^ T2) averaged from 5 brain regions (cases 1–18), two regions (cases 19 and 21) or available in one brain region (case 20)

^b^620 sCJD cases examined

^c,d^Cases identified ^b^before or ^c^after retrospective histopathological examination. ^e^Expressed as mean ± SD

^f^P < 0.03 (Student’s *t*-test)

No statistical differences were found when comparing gender, race, age at onset, and disease duration

### Brain sampling for western blot analysis

For molecular studies, frozen brain tissue from 14 p-CJD cases (cases 1–8, 10, 13–15, 17 and 18, Table 1) was sampled from five brain regions that included the frontal and occipital cortices, putamen, thalamus, and cerebellum. However, in three p-CJD cases, only the frontal cortex (case 20) or frontal cortex and cerebellum (cases 19 and 21) were available. Four cases underwent a more extensive sampling with 13 brain regions assessed (cases 9, 11, 12 and 16) that included the three gyri of the frontal lobe, the temporal, occipital, parietal and entorhinal cortices, as well as the hippocampus, caudate nucleus, putamen, anterior thalamus, midbrain, and cerebellum. In nine sCJDMM1 and five sCJDMM2 controls, we sampled nine brain regions: frontal, visual and non-visual cortices, hippocampus, entorhinal cortex, putamen, thalamus, midbrain, and cerebellum.

### Brain homogenate preparation and proteinase K digestion

**Fig. 1:**
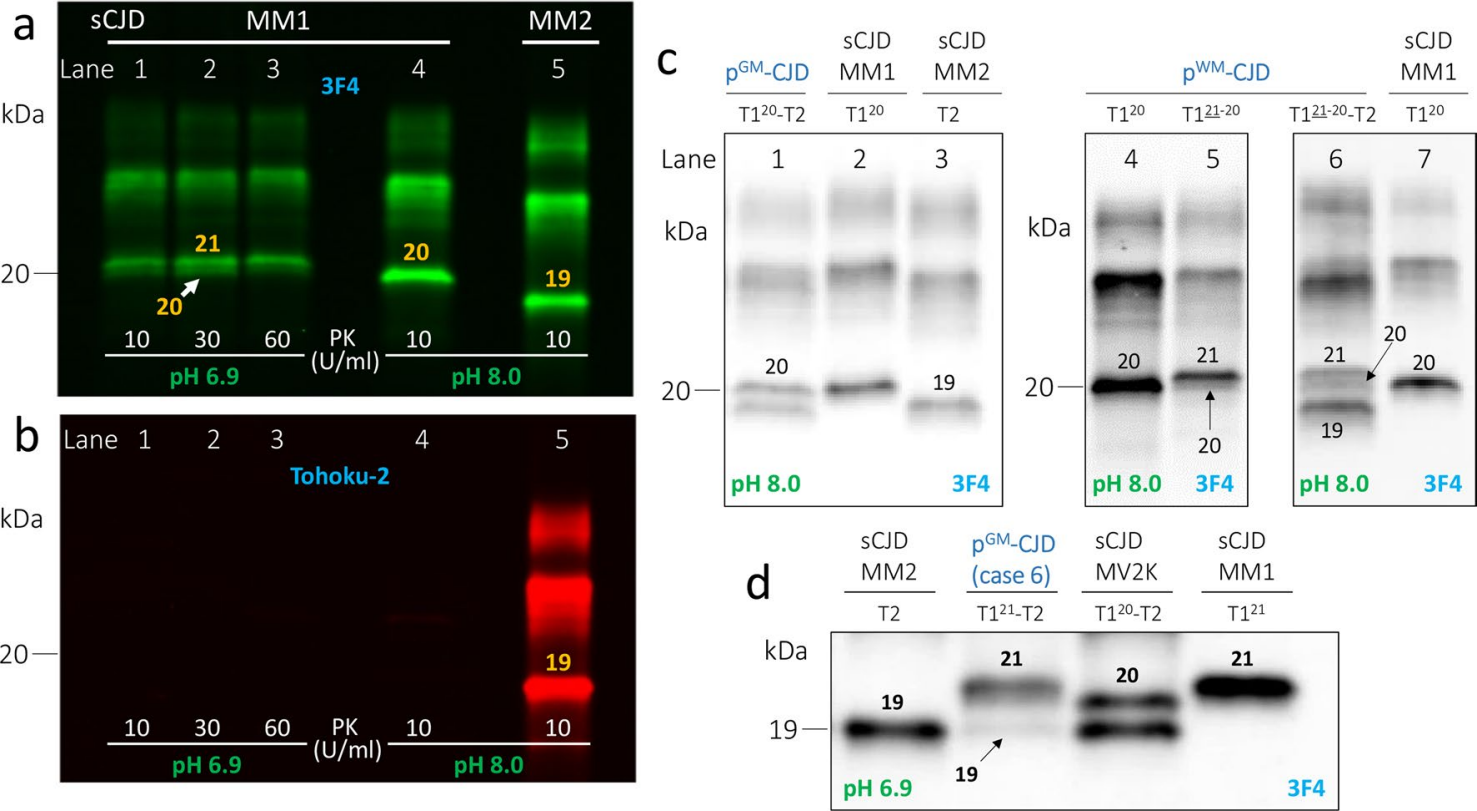
Buffer pH effect on PK-resistant PrP^D^ (resPrP^D^) gel mobility, and western blot (WB) profiles of resPrP^D^ in p-CJD subtypes. Near-infrared LICOR (**a**–**c**) and chemiluminescence (**d**). **a**, **b**: Brain homogenates (S1) from the frontal cortex of sCJDMM1 (lanes 1–4) were prepared in LB100 pH 6.9 or 8.0, digested with different PK concentrations, and probed with 3F4 (**a**) and tohoku-2 (**b**). **c** and **d**: S1 (frontal cortex) were prepared in LB100 pH 8.0 (**c**) or pH 6.9 (**d**), and were digested with 10 U/ml (**c**) or 100 U/ml (**d**) PK. **a** The WB profile of the unglycosylated isoform of resPrP^D^ T1 appears as a major fragment of ~ 21 kDa (21) (PK 10–60 U/ml) and a thinner lower band of ~ 20 kDa (20) at pH 6.9. PrP^D^ T1 appears as a single band of ~ 20 kDa at pH 8.0. Gel mobility of resPrP^D^ T2 of sCJDMM2 is ~ 19 kDa (19) at pH 8.0 (lane 5); T2 is not affected by the change of buffer pH (data not shown). **b** Tohoku 2 immunoreacts only with resPrP^D^ T2 (lane 5). **c** Samples were prepared from the putamen (lanes 1, 5 and 6) and frontal cortex (lanes 2–4 and 7). In p^GM^-CJD, the unglycosylated resPrP^D^ T1 appears as a single fragment of ~ 20 kDa co-existing with T2 (lane 1, T1^20^-T2). T1^20^ and T2 from sCJDMM1 (lanes 2 and 7) and -MM2 (lane 3) are used as controls. In p^WM^-CJD, resPrP^D^ T1 migrates as a single band of ~ 20 kDa (lane 4, T1^20^) or as a doublet of ~ 21–20 kDa with prominent ~ 21 kDa fragment (lane 5, T1^21−20^). The ~ 21–20 kDa doublet of p^WM^-CJD co-exists with a ~ 19 kDa fragment (T2) (lane 6, T1^21−20^–2). **d** At buffer pH 6.9, T1 and T2 of p^GM^-CJD migrate to ~ 21 kDa and ~ 19 kDa, respectively (T1^21^-T2). T1 of sCJDMM1, T2 of -MM2 and T1-2 of -MV2K controls migrate to ~ 21 (T1^21^), ~ 19 kDa, and as ~ 20–19 kDa doublet (T1^20^-T2), respectively. For simplicity, only the unglycosylated isoform of resPrP^D^ is shown. Numbers atop each unglycosylated resPrP^D^ band indicate the relative molecular weight

Frozen brain tissue from all p-CJD, three sCJDMM1 and three sCJDMM2 cases were homogenized in 1X DPBS to generate a 20% (wt/vol) brain homogenate (BH), which was diluted with an equal volume of 2X lysis buffer (LB) 100 (1X LB100: 100 mM NaCl, 10 mM EDTA, 0.5% NP-40, 0.5% sodium deoxycholate, 100 mM Tris–HCl, pH 8.0), and incubated on ice for 30 min (min). After centrifugation at 1000×*g* (5 min, 4 °C), the supernatant (S1) was collected into a new test tube. Brain tissue from four cases (9, 11, 12 and 16, Table 1) was homogenized as indicated above, but also homogenized directly with 1X LB100 pH 8.0 to make a 10% BH. All BH prepared in LB100 pH 8.0 were digested with 10 Units/ml (U/ml) proteinase K (PK) for 1 h at 37 °C with constant agitation [PK specific activity was 48 U/mg at 37 °C, with 1 U/ml equal to 20.8 μg/ml PK]. Cases 1–6 (Table 1), one each of sCJDMM1, sCJDMM2 and sCJDMV2K were also prepared in 1X LB100 pH 6.9 and incubated with 100 U/ml (~ 2 mg/ml) PK (Figs. 1 and S1). The enzymatic reaction was stopped with the addition of 3 mM PMSF. Finally, samples were mixed in an equal volume of 2X Laemmli buffer (6% SDS, 5% β-mercaptoethanol, 20% glycerol, 4 mM EDTA, 125 mM Tris–HCl, pH 6.9) and denatured (100 °C, 10 min).

### Methanol-chloroform-water precipitation of PrP and removing of N-linked oligosaccharides by N-Glycosidase F

Ten percent (wt/vol) BH of p-CJD (cases 9, 11, and 12), generated from the cerebellar white matter using 1X LB100 (pH 8.0), were precipitated in methanol, chloroform and water as previously described [70]. BH were treated with 5 U/ml PK (1 h at 37 °C), the reaction was stopped with 3 mM PMSF, and then mixed with an equal volume of 2X Laemmli buffer and denatured (100 °C, 10 min). For deglycosylation of PrP, denatured proteins were incubated with N-Glycosidase F (PNGase F) as previously described [9].

### Western blot analysis 

Denatured proteins from all p-CJD and 6 sCJD controls cases were loaded onto 15% Criterion™ Tris–HCl precast gels (W × L: 13.3 cm × 8.7 cm). Proteins were then transferred onto Immobilon-FL PVDF membranes, blocked with Odyssey blocking buffer for 1 h and probed with primary antibodies (3F4 and tohoku-2 at 1: 10,000 dilution; 1E4 and 12B2 at 1 µg/ml and 0.2 µg/ml, respectively) [8, 14]. Membranes were washed with 1X DPBS containing 0.1% of Tween 20 (1X DPBS-T) and probed with secondary antibodies IRDye 680RD goat anti-rabbit IgG (1: 10,000) and IRdye 800CW goat anti-mouse IgG (1: 10,000) (LICOR Biosciences). After washing in 1X DPBS-T, membranes were scanned using the Odyssey infrared imaging system (LICOR Biosciences) to PrP bands visualization. Denatured proteins from four p-CJD (cases 9, 11, 12, and 16), nine sCJDMM1 and five sCJDMM2 cases were also loaded onto 15% Tris–HCl SDS–polyacrylamide gels (W × L: 20 cm × 20 cm; Bio-Rad PROTEAN® II xi cell system) and visualized by chemiluninescence as previously described [10].

### Brain distribution of resPrP^D^ types

The brain distribution of p-CJD resPrP^D^ T1 and T2 was determined by dividing the relative amount of resPrP^D^ of each brain region to the total amount of resPrP^D^ (i.e., the sum of resPrP^D^ of five brain regions: frontal and occipital cortices, putamen, thalamus and cerebellum). Each point of the profile is expressed as a mean ± standard error of the mean (SEM).

### Conformational solubility and stability assay (CSSA) of resPrP^D^

Supernatants (S1) were digested with 10 U/ml PK for 1 h at 37 °C. Enzymatic reaction was stopped with 3 mM of PMSF. For CSSA, PK-treated S1 was incubated with equal volumes of varying concentrations of GdnHCl (i.e., 0, 0.5, 1, 1.5, 2, 3, and 4 M) for 1 h at 37 °C. Samples were then centrifuged at 18,000 × g (30 min, 4 °C). Supernatants were discarded and pellets were re-suspended in 2X Laemmli buffer. Samples were briefly sonicated and denatured again (100 °C, 10 min). Before performing CSSA, we measured signal intensity of resPrP^D^ at GdnHCl 0 M, and adjusted the loading volumes to have the same signal intensity in all cases [14]. A dose–response equation was used to fit the seven GdnHCl points. GdnHCl_1/2_ index values, expressed as mean ± SEM, were obtained with GraphPad Prism 9.5.0. In CSSA experiments, resPrP^D^ was detected by 3F4 and extracted from the cases listed below:

**Table:** 

Prion disease	PrP^D^ type	FC (n)	OC (n)	Put (n)	TH (n)	Crbl (n)
p^GM^-CJD	T1^20^	2	1	2	1	3
	T2	1	–	1	1	–
p^WM^-CJD	T1^20^	2	1	–	–	–
	T1^21^	–	–	3	–	–
	T2	1	1	2	–	–
sCJDMM1	T1^20^	2	–	3	–	–
sCJDMM2	T2	3	–	–	–	–

*FC* frontal cortex (cx), *OC* occipital cx, *Put* putamen, *TH* anterior thalamus, *Crbl* cerebellum, (*n*) number of cases assessed.

### PK-titration assay

Twenty percent (wt/vol) BH prepared in 1X DPBS were mixed with equal volume 2X LB100 (pH 8.0) and incubated on ice for 30 min. Samples were centrifuged at 1000×*g* (5 min, 4 °C), and supernatants were collected and incubated with PK 0.6, 2.5, 5, 10, 40 and 160 U/mL (1 h, 37 °C). The proteolytic reaction was stopped with 3 mM PMSF; samples were then mixed with an equal volume of 2X Laemmli buffer and denatured (10 min, 100 °C). Samples were incubated with a fivefold volume excess of pre-chilled methanol (2 h, − 20 °C), and centrifuged at 18,000×*g* (30 min, 4 °C). Pellets were re-suspended in 2X Laemmli Buffer, briefly sonicated, and denatured (5 min, 100 °C). As in the CSSA, the amount of PK-resistant PrP^D^ was normalized to have a similar signal intensity in each case. The signal intensity of resPrP^D^ was then measured as previously described [14]. For both T1 variants, PK points were best fitted as previously described [14]. Each point of the profile is expressed as a mean ± SEM.

### RT-QuIC analysis of 10% brain homogenates

RT-QuIC testing of serially diluted 10% brain homogenates was performed as previously described [53]. In brief, the reaction mix was composed of 10 mM phosphate buffer (pH 7.4), 300 NaCl, 0.1 mg/ml bank vole 23–230 rPrP^Sen^, 10 μM thioflavin T (ThT), 1 mM ethylenediaminetetraacetic acid tetrasodium salt (EDTA), and 0.001% SDS. The reaction mix (98 μL per well) was loaded into each well of a black 96-well plate with clear bottom (Nunc) and seeded with 2 μl of BH dilutions. The plate was then sealed with a plate sealer film (Nalgene Nunc International) and incubated at 42 °C in a BMG FLUOstar Omega plate reader alternating cycles of 1 min shaking (700 rpm double orbital) and 1 min rest during the indicated incubation time. ThT fluorescence measurements (450 ± 10 nm excitation and 480 ± 10 nm emission; bottom read) were taken every 45 min. Reactions were classified as RT-QuIC positive base on criteria previously described for RT-QuIC analyses of brain specimens [53]. Kinetic plots are average of a total of 8 reaction wells from 2 independent experiments.

### Immunohistochemistry, thioflavin S, and periodic acid–Schiff (PAS) staining

Eight-micron formalin-fixed paraffin-embedded tissue sections were deparaffinized and rehydrated before being microwaved in a 1.5 mM HCl solution for antigen retrieval. Sections were immersed in two baths of 1X TBS containing 0.05% Tween-20 (1X TBS-T) for 15 min each. Endogenous peroxidase was blocked using a 2.4% hydrogen peroxide (H_2_O_2_) solution for 10 min, after which sections were washed in 1X TBS-T, blocked with 10% normal goat serum, and incubated with the antibody 3F4 (1: 1000) for one hour. Sections were washed in 1X TBS-T and incubated with the Dako Envision + System HRP Labelled Anti-Mouse (Agilent, Santa Clara, CA) for 30 min. After washing with 1X TBS-T, sections were incubated with Envision Flex DAB (Agilent) for PrP visualization. Finally, tissue was counterstained with hematoxylin and bluing reagents and dehydrated. Fourteen H&E sections were examined for histopathological assessment: frontal, occipital, temporal, parietal and entorhinal cortices, as well as the hippocampus, anterior basal ganglia, anterior thalamus, hypothalamus, midbrain, pons, medulla, cerebellar vermis and hemispheres. For PrP immunohistochemistry (IHC), 9 regions were stained (frontal, temporal, occipital and entorhinal cortices, hippocampus, basal ganglia, midbrain, cerebellar vermis and hemispheres). Severity of plaque pathology was rated on a scale of 0–3 (0, not detectable; 1, mild: 2, moderate; 3, severe). Size of PrP plaques, expressed as diameter, was measured in the cerebellum of five p^GM^-CJD and eight p^WM^-CJD cases.

We performed Amyloid-β (Aβ) and hyperphosphorylated tau (p-tau) IHC using 4G8 (1: 2,000) and AT8 antibodies (1: 200), respectively, on five brain regions that included the hippocampus, frontal, temporal, occipital and entorhinal cortices. For staining with thioflavin S, deparaffinized sections were stained in thioflavin S (7 min), washed in 80% alcohol (3 times), dehydrated in ethyl alcohol, cleared in xylene, and cover slipped with mounting medium for fluorescence (Vectashield, Vector Laboratories).

PAS staining was performed by immerging 4 µm-thick slides in periodic acid followed incubation with the Schiff’s reagent.

### Electron microscopy

Twenty-micron thick sections were used for electron microscopy as previously described [44].

### Transgenic mice expressing wild type or glycan-free human PrP and transmission study

Two transgenic (Tg) mouse models were used. The first model expresses the wild-type (WT), fully glycosylated, human PrP^129M^ (TgHuPrP^Gly+/+^) in the FVB-NJ strain PrP-KO background at ~ twofolds the normal murine PrP brain levels [39, 52]. The second model carries the WT human PrP^129M^, and the mutated human PrP due to substitution of the two Asn residues at positions 181 and 197 with Gln which eliminates both N-linked glycosylation sites (TgHuPrP^Gly+/–^) (Figs. S2 and S3). In this study, TgHuPrPGly^+/+^ mice refer to the Tg40h mice; TgHuPrPGly^+/–^ mice refer to progenies of breeding between Tg40h (TgHuPrP^Gly+/+^) and TgNN6h (TgHuPrPGly^−/−^) mice that contain one wild type human PrP-129 M allele from the Tg40h mice and one mutant human PrP181Q/197Q allele from the TgNN6h mice [27], leading to expressing of both wild-type glycosylated human PrP-129 M protein and mutant glycan-free human PrP181Q/197Q protein. Mice were inoculated into the left parietal cortex with 30 µl of 1% BH obtained from the putamen of one p^WM^-CJD case (case 12), and the putamen of one sCJDMM1 case, according to previously described procedures [38]. A total of 12 mice were used: four TgHuPrP^Gly+/+^ and eight TgHuPrP^Gly+/–^. After inoculation, mice were examined daily for symptoms of prion disease (e.g., waddling gait, tail plasticity, coarse coat, and bradykinesia). Two to three days after the appearance of clinical disease, mice were culled and the brains used for biochemical and histopathological examination as previously described [12].

### Image acquisition, densitometric analysis and statistical tests

All microphotographs were taken with Leica DFC 425 digital camera mounted on a Leica DM 2000 microscope, except for microphotographs of thioflavin S stained section which were taken with an Olympus IX71. Diameter of PrP plaques was measured with Image-Pro Plus 7.0 (Media Cybernetics, Inc.). Statistical significance was determined using a Student’s t-test (two-tailed) for the PK-titration assay and brain distribution of resPrP^D^ types (GraphPad Prism 9.5.0). Statistical significance was determined by one-way ANOVA for the CSSA experiments.

### Clinical evaluation

Medical records are requested when cases are submitted to the NPDPSC for neuropathologic examination, however, the amount, quality, and homogeneity of the obtained data is variable. The legal next of kin completes an autopsy consent form that includes information on recognized and possible acquired prion disease risk factors. Data were collected on demographics such as gender, race/ethnicity, age at disease onset, and disease duration. Medical records, including past medical and surgical histories as well as other risk factors for the development of iatrogenic prion disease, clinical symptoms, family history, and diagnostic test results were obtained and reviewed by a clinician. These data were not available in all cases.

### Genetic analysis of human and mouse

DNA was extracted from frozen human brain sections in all cases examined. Genetic analysis was carried out to rule out mutations in the PrP gene as well as to determine the polymorphism at codon 129 of *PRNP*. Genetic analysis was performed as previously described [55, 58]. In mice, the extracted DNA was used as template for polymerase chain reaction (PCR) to amplify part of the PRNP exon 2 using HRM-F/HRM-R primers [7, 38]. These primers, plus INT5 and INT3 [7, 38] were used for bidirectional sequencing of PCR products using Applied BiosystemsTM BigDyeTM Terminator v3.1 cycle Sequencing Kit on a 3500 Genetic Analyzer. The final sequencing results were used to analyze the open reading frame (ORF) from + 76 bp through + 762 bp, stop codon of PRNP exon 2.

## Results

### Prevalence of cases with PrP plaques in sCJD-129MM.

We performed a retrospective examination of 620 confirmed sCJD cases—with MM genotype at PrP-codon 129 (sCJDMM)—for the presence of PrP plaques in the cerebellum. All cases were diagnosed at the NPDPSC and included 463 -MM1, 48 -MM2 and 109 -MM1-2. From this assessment, we found PrP plaques in 14 cases (2.2%). Furthermore, PrP plaques (p) occupied either the gray matter (p^GM^-CJD), and were mostly confined in the cerebellar cortex (*n* = 7) or populated the white matter (p^WM^-CJD) and were seen all throughout the brain (n = 7). In addition to these 14 p-CJD cases identified within the large sCJDMM cohort, seven p^WM^-CJD cases were identified independently (Table 1).

Prior to re-characterization, the prevalence of cases harboring: (i) resPrP^D^ T1 only (i.e., T2 could not be detected in any of the brain regions assessed) was 14% in p^GM^-CJD and 71% in p^WM^-CJD; (ii) resPrP^D^ T2 only was 14% in p^GM^-CJD and 0% in p^WM^-CJD (*P* < 0.03); (iii) co-existing resPrP^D^ types 1 and 2 (T1-2 = T1 and T2 co-existing in one or more brain regions) was 57% in p^GM^-CJD and 14% in p^WM^-CJD (P > 0.05).

### Case re-classification

We carried out a re-characterization of resPrP^D^ types in all p-CJD cases to identify distinct signatures of resPrP^D^ in brain regions that were not analyzed during routine western blot analysis at the NPDPSC.

A minimum of five brain regions were assessed in 86% of p-CJD cases. Exceptions to this rule were three cases with less than five brain regions available and four cases with a plethora of brain regions (13 regions each) (Table 1). By increasing the number of brain region assessed and antibodies employed, prevalence of resPrP^D^ T1-2 increased from 57 to 100% in p^GM^-CJD cases, and from 14 to 64% in p^WM^-CJD, while prevalence of resPrP^D^ T1 decreased from 71 to 36% in p^WM^-CJD (Table 1).

The percent representation of T2 out of total resPrP^D^, measured as a mean value of all brain regions assessed in each case, ranged from 3 and 98% in p^GM^-CJD, and from 2 to 60% in p^WM^-CJD. The percentage of T2 averaged from all cases was 44% and 14% in p^GM^-CJD and p^WM^-CJD, respectively (*P* < 0.05). In the cerebellum, the percentage of T2 was low (33%) in p^GM^-CJD and absent altogether in p^WM^-CJD (Table 1).

### Buffer pH affects gel mobility of resPrP^D^ T1

It has been shown that the buffer pH has a strong effect on PK cleavage of PrP^D^ and, in turn, the mobility of resPrP^D^ [9, 50]. Brain tissue from a sCJDMM1 case was homogenized in lysis buffer with 100 mM Tris at pH 6.9 or 8.0, and digested with 10, 30 and 60 U/ml PK at pH 6.9 or 10 U/ml PK at pH 8.0. The unglycosylated isoform of resPrP^D^ T1 obtained with lysis buffer pH 6.9 migrated as a doublet ~ 21–20 kDa (T1^21−20^), with ~ 21 kDa being the predominant band (T1^21−20^) at any PK concentration (Fig. 1a). On the other hand, T1 obtained with lysis buffer pH 8.0 migrated as a single band of ~ 20 kDa (T1^20^). These results confirm that the buffer pH modulates resPrP^D^ T1 gel mobility. Furthermore, T1 obtained at either pH was not detected by tohoku-2, a conformational antibody that immunoreacts only to T2 (Fig. 1b) [36]. Unlike T1, PrP^D^ T2 was not influenced by the buffer pH and was detected by both 3F4 and tohoku-2 antibodies (Fig. 1a, b) [50]. Overall, the ratio of di-, mono-, and un-glycosylated resPrP^D^ isoforms was typical for sCJD, and none of the p-CJD cases showed a predominance of the di-glycosylated resPrP^D^ isoform that characterizes vCJD, BSE, and CWD.

### T1^21^ and T1^20^ variants of p-CJD

We found that the use of lysis buffer at different pH discriminates between resPrP^D^ T1 variants associated with p^GM^- and p^WM^-CJD. Using lysis buffer pH 8.0, all p^GM^-CJD cases harbored T1^20^ as in sCJDMM1 (Fig. 1c). Additionally, in most brain regions of all p^GM^-CJD cases, T1^20^ co-existed with a ~ 19 kDa fragment that matched the gel mobility of sCJDMM2 (Fig. 1c). Unlike with p^GM^-CJD, T1^21−20^ populated the subcortical regions of most p^WM^-CJD (Fig. 1c and Table 2). These differences between p^GM^-CJD and p^WM^-CJD would not have been detected at buffer pH 6.9, as T1^21−20^ would be observed in both p-CJD subtypes and in sCJDMM1 (Figs. 1d and S1). The PK cleavage site responsible for the generation of T1^20^ is a downstream from glycine 82, and may correspond to tryptophan 89 (Fig. S4) [51, 58].

**Table 2:** Molecular and histopathological features of Us p^GM^- and p^WM^-CJD subtypes

Case number	Age at onset (years)	Disease duration (months)						PrP immunostaining pattern				
			PK-resistant PrP^D^					Plaques			Coarse	“Brush stroke-like”
					MM2-like CC (%) ^d^	CC ^e, f^	Subc ^e, f^	Cerebellum				
								Gray matter		White matter		
			T1^21^ (%) ^a^	T2 (%) ^b, c^				Mol. L	Grl. L. ^g^		Crbl Mol. L	Crbl Mol. L
p^GM^-CJD												
1	63	12	0	98	90	0	0	3	1	0	+	–
2	73	4	0	89	53	0	0	2	0.5	0	+	+
3	68	24	0	45	90	0	0	1	0.5	0	+	+
4	71	2	0	29	20	0	0	1.5	1	0	–	+
5	57	2	0	26	40	0	0	2	1	0	–	+
6	60	2	0	16	43	1	0	3	2	0.1	–	+
7	83	2	0	3	5	0	0	0	1	0.1	–	+
Mean ± SD	68 ± 9	7 ± 8	0	44 ± 36	49 ± 32	0.1 ± 0.4	0	1.8 ± 1.1	1 ± 0.5	0.03 ± 0.05	43^h^(3/7)^i^	86^h^(6/7)^i^
p^WM^-CJD												
8	72	9	67	60	80	0.5	1.5	0	0	2	+	+
9	54	11	55	50	57	1.5	2	0	0	2	+	–
10	69	7	23	40	37	0.5	2	0	0	2	+	+
11	52	11	65	24	20	1	1.5	0	0	2	–	+
12	59	7	25	8	10	2 ^j^	1.5	0	0	1	+	+
13	75	1	62	5	5	0.5	1	0	0	1	–	+
14	51	7	52	5	0	0.5	1	0	0	1	–	+
15	72	9	27	4	30	0.5	3	0	0	2	+	+
16	57	13	46	2	0	3 ^j^	3	0	0	3	–	+
17	62	7	56	0	0	2	3	0	0	2	–	+
18	44	3	22	0	0	1.5	1.5	0	0	0.5	–	+
Mean ± SD	61 ± 10	8 ± 3.5	45 ± 18	18 ± 22	22 ± 27	1.2 ± 0.8	1.9 ± 0.8	0	0	1.7 ± 0.7	45^h^(5/11)^i^	91^h^(10/11)^i^
*P* value	NS	NS	< 0.0001	NS	NS	< 0.007	< 0.0001	< 0.005	< 0.002	< 0.0001	NS ^k^	NS ^l^

^a^Percentage of PK-resistant PrP^D^ (resPrP^D^) T1^21^ out of total resPrP^D^ T1 (total T1 = T1^21^ + T1^20^) averaged from subcortical (Subc) regions (putamen and thalamus)

^b, c^Data obtained from the ^b^ cerebral cortex (frontal and occipital cortices)

^c^Expressed as percentage of resPrP^D^ type 2 (T2) out of total resPrP^D^ (total resPrP^D^ = resPrP^D^ T1 + resPrP^D^ T2)

^d^Data expressed as percentage of the surface of cerebral cortex (frontal, temporal and occipital cortices) occupied by coarse and/or perivacuolar IP

^e, f^It refers to ^e^ gray and white matter (cases 1–7) or ^f^ white matter (cases 8–18)

^g^It includes the Purkinje cell layer

^h^Expressed in %

^i^Cases with the feature listed/total cases examined

^j^Rare PrP plaques affecting layer VI of the gray matter. PrP plaque severity was scored on a 0–3 scale (0, absent; 1, mild; 2, moderate; 3, severe). Statistical significance was calculated using Student’s *t*-test

^k^Fisher’s exact tes

^l^Chi-square test

*Mol. L.* molecular layer, *Crbl* cerebellum, *Grl. L.* granular layer, *NS* not significant

### Prevalence and brain distribution of T1 and T2

**Fig. 2:**
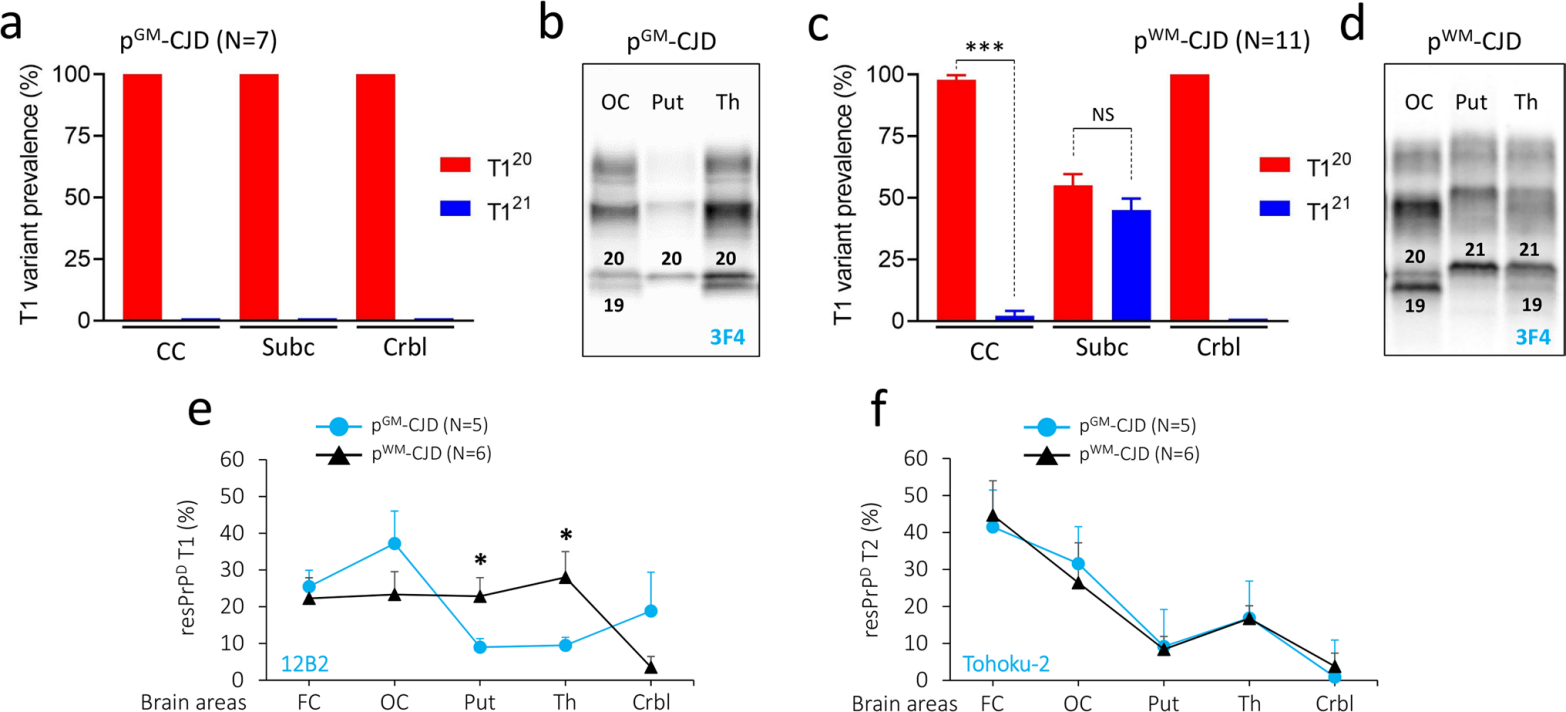
Topographic distribution of resPrP^D^ T1^21^, T1^20^ and T2. **a** In p^GM^-sCJD, resPrP^D^ T1^20^ is the only T1 variant detected; CC: cerebral cortex; Subc: subcortical regions; Crbl: cerebellum. **b** WB representation of T1^20^-T2 in the occipital cortex (OC) and thalamus (Th), and T1^20^ in the putamen (Put). **c** Unlike p^GM^-sCJD, T1^21^ is well represented in Subc of p^WM^-sCJD **d**: WB depicting T1^21−20^ in Put, T1^21−20^-T2 in Th, and T1^20^-T2 in OC. Numbers above unglycosylated resPrP^D^ bands indicate the relative molecular size; *P < 0.05; ***P < 0.0001 (Student’s t-test). **e**, **f** Amount distribution of resPrP^D^ assessed by the type-discriminatory antibodies 12B2 (to T1, **e**) and tohoku-2 (to T2, **f**) antibodies. **e** In p^GM^-sCJD, T1^20^ amount in CC is higher than in Subc and Crbl, whereas in p^WM^-sCJD, T1 amount is equally distributed in CC and Subc. *P < 0.05 (Student’s *t*-test). **f** The amount of T2 is virtually identical in p-CJD subtypes. Each point of the profile is expressed as mean ± SEM

We measured the ratio of unglycosylated resPrP^D^ T1^21^ and T1^20^ fragments in the various brain regions of p-CJD cases. While T1^21^ was absent or significantly underrepresented in the brain of the p^GM^-CJD cases, T1^21^ was the predominant T1 variant in the subcortical regions of six p^WM^-CJD cases (55%) at pH 8.0 (Figs. 1c and 2a–d, and Table 2). In the remaining five cases, the T1^21^:T1^20^ ratio was ~ 30%:70%.

Using PrP^D^ type-selective antibodies, 12B2 for T1 (12B2 does not discriminate between T1^21^ and T1^20^ variants) and tohoku-2 for T2, we determined the amount of T1 and T2 resPrP^D^ in various brain regions[14]. Notably, the amount of T1 differed in the two p-CJD subtypes, and it was greater in the cerebral cortex than in subcortical regions in p^GM^-CJD, but not in p^WM^-CJD, where the amount of T1 was comparable in the cerebral cortex and the subcortical regions (*P* < 0.05) (Fig. 2e). T2 distribution was virtually identical in p^GM^- and p^WM^-CJD subtypes (Fig. 2f).

We also determined the western blot profile of the PrP extracted from the deep white matter of the cerebellum in p^WM^-CJD and sCJDMM1 and -MM2 cases. Western blot profiles of total PrP (PrP^C^ + PrP^D^) did not show differences between p^WM^-CJD and sCJD (Fig. S5 a, b). Following incubation with PK and with peptide N-glycosidase F to remove the glycans, the unglycosylated resPrP^D^ isoform appeared as single band of ~ 20 kDa in p^WM^-CJD and sCJDMM1. Perhaps due to the presence of lipids, resPrP^D^ could not be fully diglycosylated, as mono- and di-glycosylated resPrP^D^ isoforms were detected at longer exposure times (Fig. S5 c).

### Biochemical features of resPrP^D^ T1 and T2

**Fig. 3:**
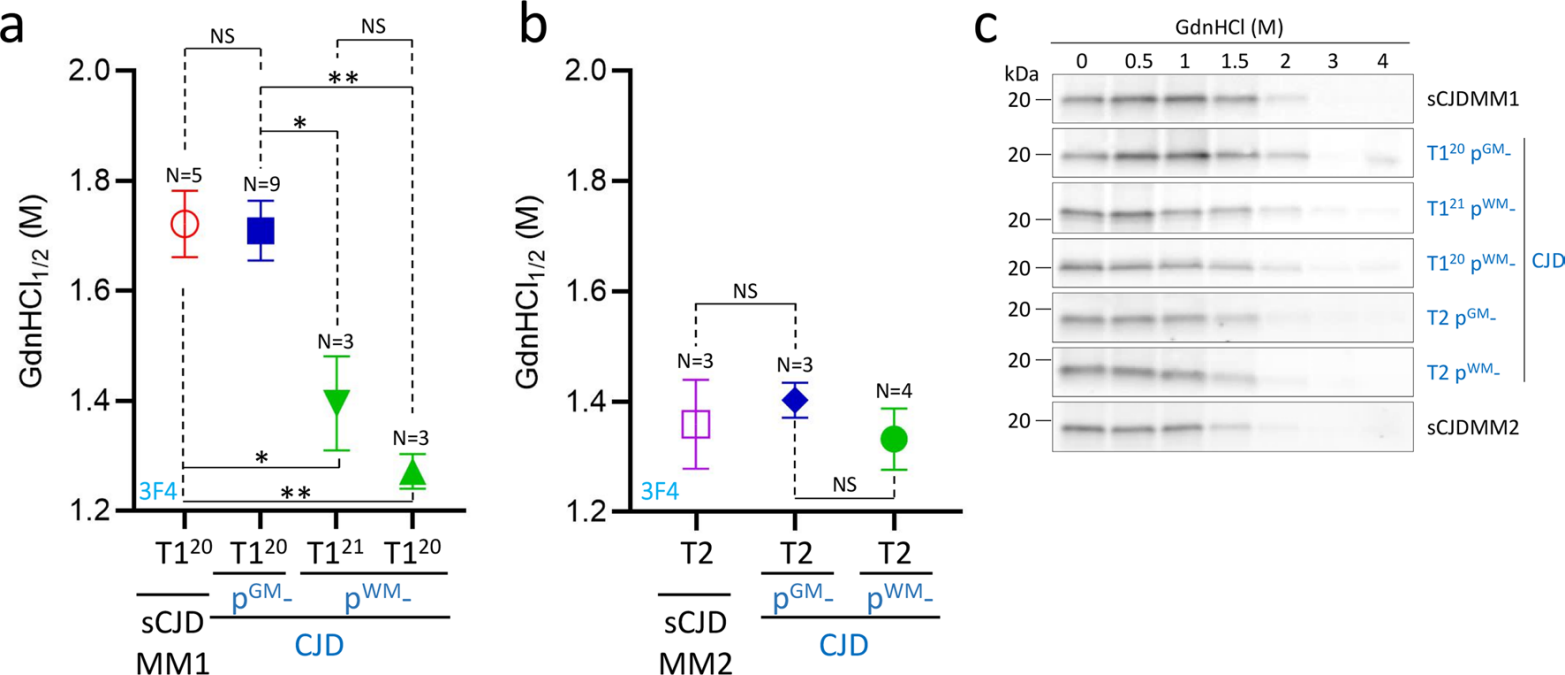
Conformational solubility and stability assay (CSSA) of resPrP^D^.** a** GdnHCl_1/2_, the amount of GdnHCl needed to solubilize 50% of resPrP^D^, is virtually identical in T1^20^ of p^GM^-CJD (1.71 M) and sCJDMM1 (1.72 M), and both differ from T1^20^ (1.27 M) and T1^21^ (1.39 M) of p^WM^-CJD. **b** GdnHCl_1/2_ index of T2 is similar in p^GM^-sCJD, p^WM^-sCJD and sCJDMM2 (1.33–1.4 M). GdnHCl_1/2_ is expressed as mean ± SEM. **c** Representative WB of T1 variants and T2 harvested from p-CJD as well as sCJDMM1 and sCJDMM2 controls. Antibody: 3F4. *P < 0.03–0.02; **P < 0.003–0.001; *NS* not significant (one-way ANOVA)

The conformational solubility and stability assay (CSSA) examines the solubility of resPrP^D^ in relation to the increasing molar (M) concentration of the denaturing agent guanidine hydrochloride (GdnHCl), in which brain samples are incubated. The GdnHCl_1/2_ index, the concentration of GdnHCl needed to solubilize 50% of the resPrP^D^, was virtually identical for T1^20^ of p^GM^-CJD and sCJDMM1 (~ 1.7 M). GdnHCl_1/2_ indexes of p^WM^-CJD T1^20^ and T1^21^ were also similar (1.27 and 1.39 M), but both differed significantly from those of p^GM^-CJD and sCJDMM1 T1^20^ (Fig. 3a, c). Unlike T1, resPrP^D^ T2 associated with p-CJD subtypes and sCJDMM2 showed similar GdnHCl_1/2_ values (Fig. 3b, c). In addition to CSSA, we carried out a PK-titration assay of T1^20^ and T1^21^ variants associated with p^WM^-CJD. The PK_1/2_ index, PK concentration required to digest 50% of PrP^D^, of T1^21^ exceeded that of T1^20^ by fourfold (PK_1/2_ T1^21^: 60 U/ml; PK_1/2_ T1^20^: 14 U/ml) (Fig. S6).

### RT-QuIC amplification of PrP^D^ in sCJD brain homogenates

**Fig. 4:**
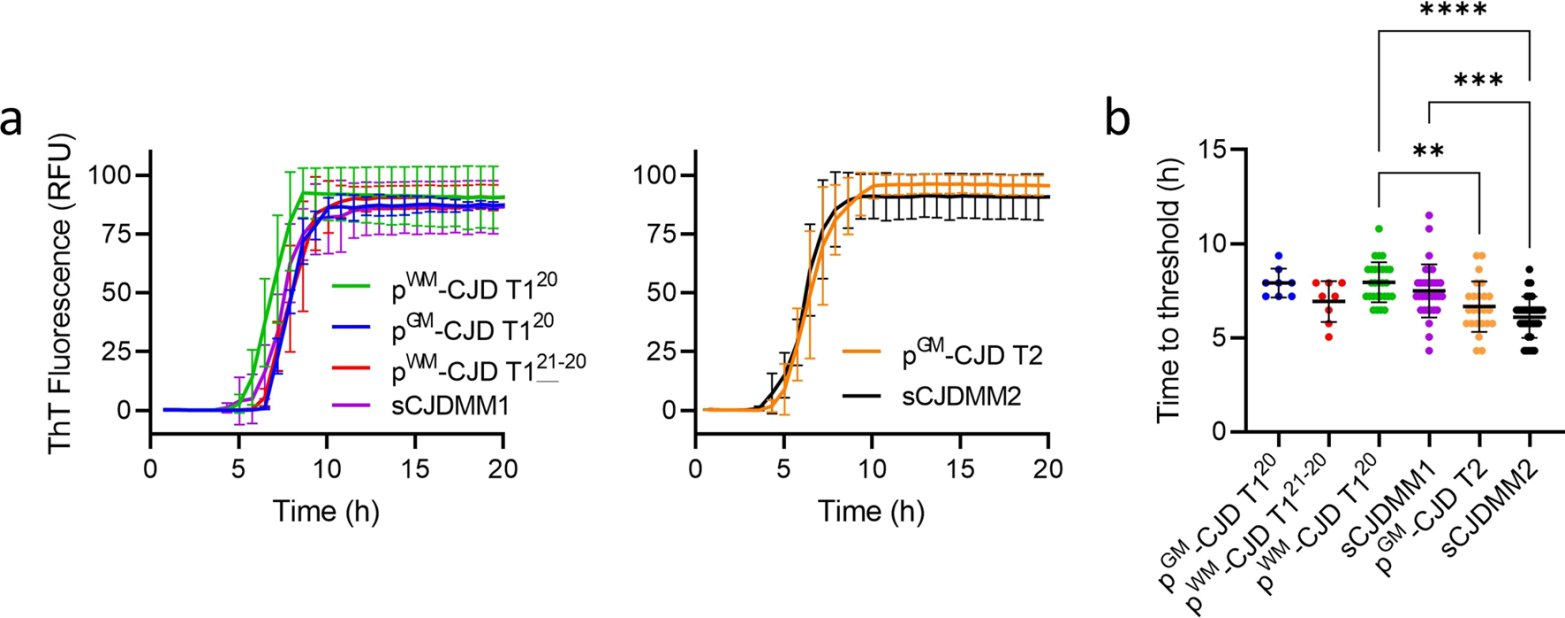
RT-QuIC amplification kinetics and time to threshold for p^WM^, p^GM^, MM1 and MM2 sCJD types. **a** Samples with predominant T1 or T2 resPrP^D^ are compared in the left and right panels, respectively. Each curve represents the mean ± SD of 8 replicate reactions seeded with 10^–4^ dilutions of brain tissue, combined from 2 independent experiments. Thioflavin T (ThT) fluorescence is shown in relative fluorescence units (RFU) as a function of the reaction incubation time. **b** Times to reaching the positivity fluorescence threshold from the experiments shown in **a**: Each symbol represents an individual reaction. **P < 0.004; ***P < 0.0002; ****P < 0.0001

To investigate the ability of the RT-QuIC to discriminate between sCJD subtypes we seeded quadruplicate reactions with 10^–4^ brain tissue dilutions from p^GM^-CJD, p^WM^-CJD, sCJDMM1 and sCJDMM2 sCJD cases (Fig. 4a). Kinetic plots showed rapid seed amplification and discrimination vs. the Alzheimer’s disease negative control, and overall similar curve profiles for all the samples (Fig. 4a). We further analyzed this data to look at time to fluorescence threshold for each replicate reaction (Fig. 4b, c) and observed a statistically significant difference in mean values between p^WM^-CJD T1^20^ and sCJDMM2 (*P* < 0.0001). We also saw a difference between sCJDMM1 and sCJDMM2 (*P* < 0.0002) and to a lesser extent between p^WM^-CJD T1^20^ and p^GM^-CJD T2 (*P* < 0.004). However, the bases for such observed differences could be multifaceted and do not necessarily reflect differences in seed structure (see Discussion).

### Prion disease histopathology

#### p^GM^-CJD subtype

**Fig. 5:**
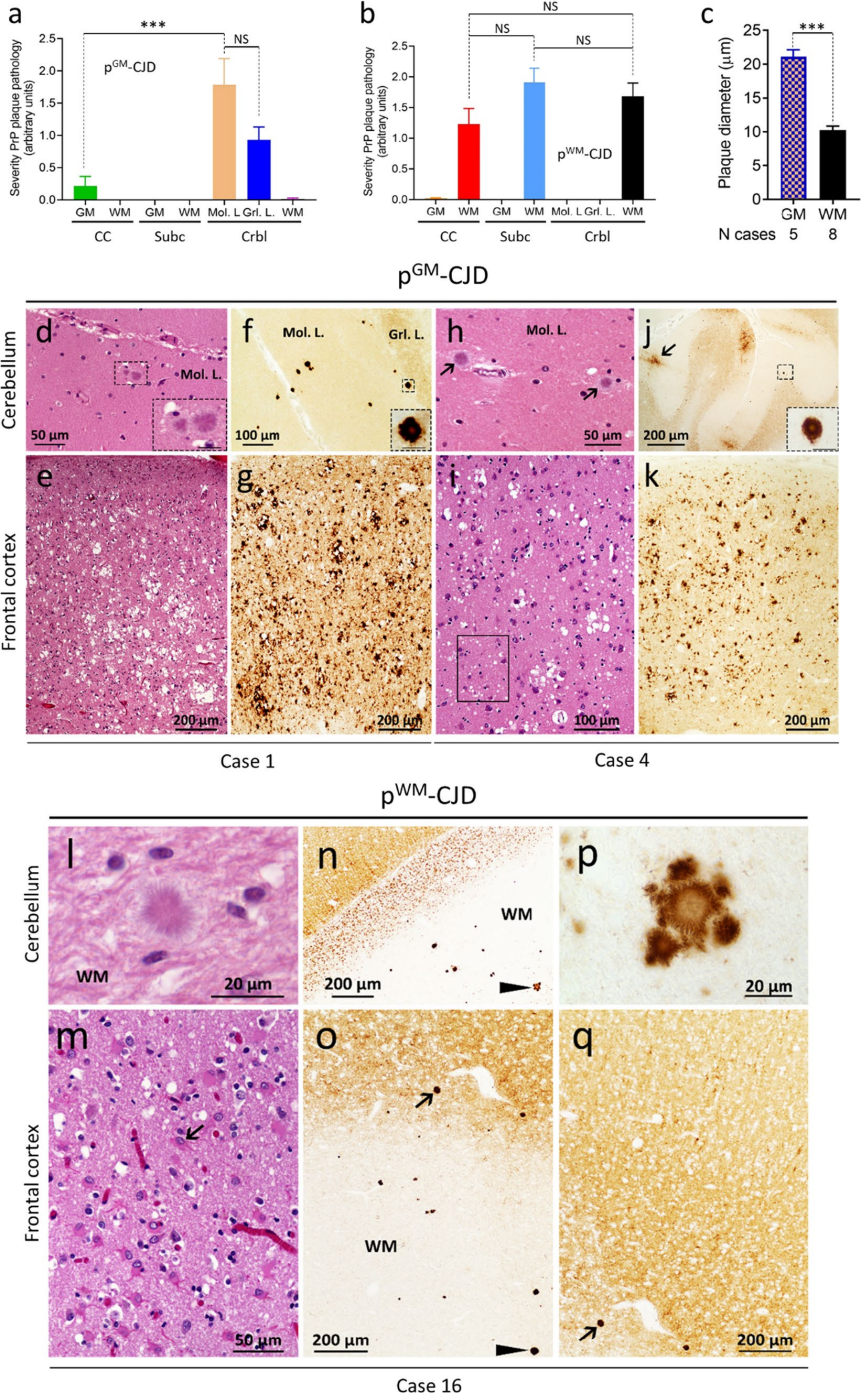
Plaque distribution and prion disease pathology.** a** In p^GM^-CJD, PrP plaques are detected in the cerebellum (Crbl) and cerebral cortex (CC). **b** In p^WM^-sCJD, PrP plaque pathology is most severe in subcortical regions (Subc) than in Crbl and CC. *GM* gray matter, *WM* white matter, *Mol. L.* molecular layer, *Grl. L.* granular layer. **c** Cerebellar plaque size is ~ twofold greater in p^GM^-sCJD than p^WM^-sCJD; ***P < 0.0001 (**a**, **c**); *NS* not significant (Student’s *t*-test). Hematoxylin–eosin, H&E, staining (**d**,** e h**,** i**, **l**,** m**) and PrP immunostaining (**f**, **g**, **j**, **k**, **n**–**q**). **d**, **h**, **l** Kuru-type plaques; inset, **d** higher magnification of the area marked by the square; arrows, **h** kuru plaques. **e**, **i** Spongiform degeneration (SD) with prominent large (**e**) or small (**i**, rectangle) vacuoles. **m**: Severe gliosis and mild SD; arrow: a reactive astrocyte.** f**, **j** PrP plaque (**f**, **j**) and brush stroke-like PrP immunostaining patterns (IP) (**j**, arrow); inset, **f** and **j**: higher magnification of a PrP plaque. **n** PrP plaques; arrowhead: plaque cluster. **p** Higher magnification of PrP plaque cluster (arrowhead in **n**). **g**, **k** Coarse and perivacuolar PrP IP. **o**, **q** PrP plaques affecting the deep WM (**o**) and layer VI of the cerebral cortex (**o**, **q**); arrow in (**o,** **q**) pointing to the same PrP plaque. Antibody: 3F4; scale bar inset in **d**, **f** and **j**: 20 µm

**Fig. 6:**
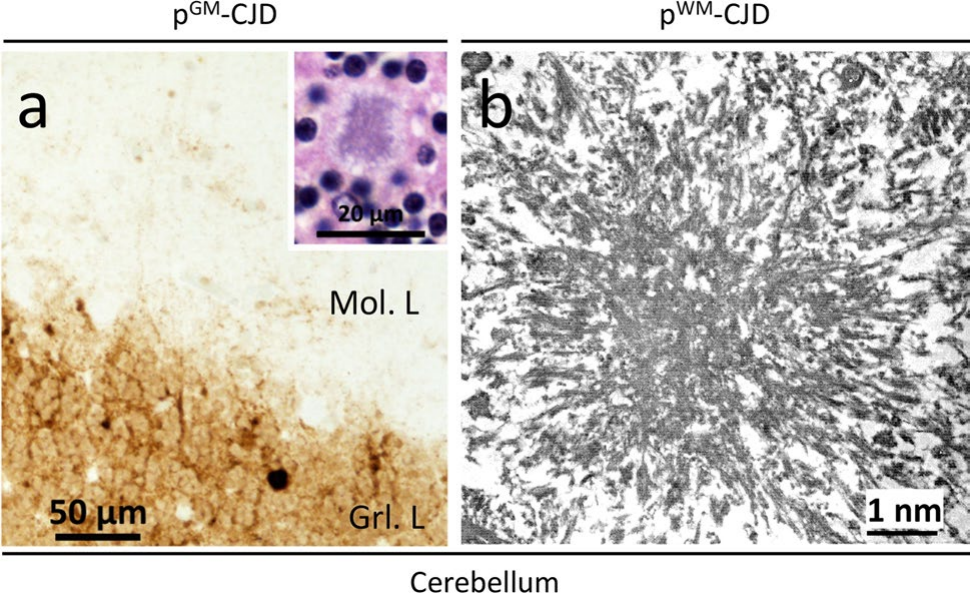
PrP immunostaining and electron microscopy. **a** PrP plaque affecting the granular layer (Grl. L.) (case 7); inset: a kuru plaque in the Grl. L.; antibody: 3F4. **b** Electron microscopy of a cerebellar, white matter, stellate kuru-type plaque (case 14)

**Fig. 7:**
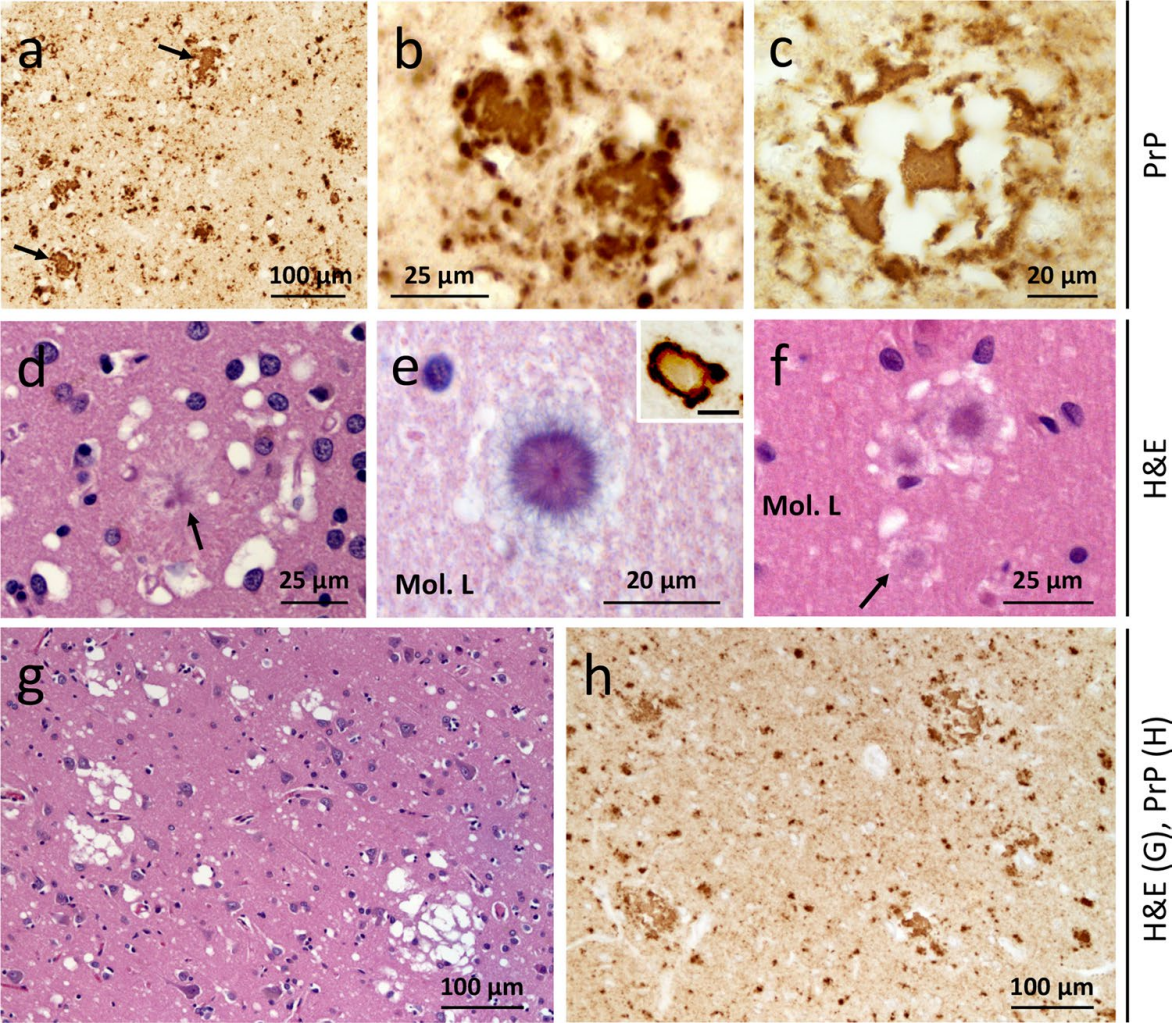
PrP plaque and plaque-like pathology of p^GM^-CJD.** a, b** Parahippocampus. **c**, **g**, **h** Frontal cortex (cx). **d** Occipital cx. **e**, **f**: Cerebellum. Case 2: (**a**–**c**); case 5: (**f**); case 6: (**d**, **e**, **g**, **h**). **a**, **b** Target-like PrP formations (arrows; not identifiable on H&E preparations) at low (**a**) and high (**b**) magnification. **c** The center of a target-like PrP core is surrounded by vacuoles and dense PrP at the periphery. **d**: A rare kuru plaque (arrow). **e** A mature kuru plaque with an eosinophilic core and radially oriented amyloid fibers; inset: a plaque with center pale and a marked rim of PrP (scale bar: 25 µm). **f** Cluster of florid plaques; arrow: a florid plaque. *Mol. L.* molecular layer. **g**, **h** Large and confluent vacuoles (**g**), and target-like PrP formations (**h**)

The pathological hallmark of this subtype is the presence of PrP plaques of the kuru-type in the cerebellar gray matter, which was prominent in the molecular layer in cases with resPrP^D^ T2 percentages ranging from ~ 16% to 98% (Fig. 5a and Table 2). In case 7, where T2 accounted for ~ 3%, PrP plaques accumulated only in the granular and Purkinje layers (Fig. 6a). Rare plaques in the white matter were observed in cases 6 and 7 (Table 2), the two cases with the most severe plaque pathology (case 6) and lowest T2 percentage (case 7). In case 5, rare florid plaques affected the cerebellum molecular layer (Fig. 7f), whereas in case 6 kuru plaques were noted in the occipital cortex (Fig. 7 d). Kuru and florid plaques were also revealed by PAS staining (Fig. S7a). In the cerebral cortex and subcortical regions, spongiform degeneration (SD) showed large and often confluent vacuoles mixed with small vacuoles, as in sCJDMM1-2 [8]. Furthermore, depending on the percentage of T2, histopathology resembled sCJDMM2 (T2 >  > T1), -MM1 (T1 >  > T2), or -MM1-2 (T1≃T2; Fig. 5e, g, i, k, and Table 2) [8]. Gliosis was typically co-distributed with SD. Following PrP immunostaining, cerebellar kuru and florid plaques were positively labeled by the 3F4 antibody (Figs. 5f, j, 6 and data not shown). In the cerebellum, coarse PrP deposits affected the molecular layer of cases with T2 amount greater than 45%, while a “brush stroke-like” PrP pattern was seen in all cases except case 1 (Fig. 5j and Table 2). The mean diameter of PrP plaques was ~ 20 µm (Fig. 5c). PrP in the cerebral cortex showed both perivacuolar/coarse and diffuse/ “synaptic” patterns in various ratios depending on the relative proportions of T1 and T2 [8]. Furthermore, target-like PrP plaque formations, not discernible on H&E preparations, were noted in the cerebral cortex of cases 2 and 6 (Fig. 7a–c).

#### p^WM^-CJD subtype

This subtype is characterized by widespread white matter PAS-reactive PrP plaques of the kuru-type (Fig. S7b). Plaques were more abundant in the subcortical regions and cerebellum than in cerebral cortex (Fig. 5b and Table 2). Cortical SD resembled that of the sCJDMM1-2 (cases 8–13, 15) or -MM1 (cases 14, 16–18) subtypes.

PrP immunostaining depicted PrP plaques throughout the brain. In two cases with the most severe plaque pathology, amyloid plaques were clustered and accumulated in cerebral cortex layer VI (Fig. 5n–q, and Table 2). The PrP plaque mean diameter was ~ 10 µm, about 2 times smaller than in p^GM^-CJD (*P* < 0.0001). Similar to p^GM^-CJD cases, coarse PrP formation in the cerebellum was seen in ~ 50% of the cases, and it was more frequent in those with higher percentages of T2. Brush stroke-like PrP was detected in almost all cases. In the cerebral cortex, mixed perivacuolar/coarse and diffuse PrP patterns were typical of cases harboring T1-2, whereas cases harboring only T1, exclusively exhibited a diffuse PrP pattern (Fig. 5q and Table 2).

#### Correlations between histotype, disease course and PrP^D^ type

In p^GM^-CJD, disease duration decreased from 13 ± 10 months to 2 months, in cases with mean T2 of 77% (cases 1–3), to 19% (cases 4–7), respectively (r = 0.36), whereas in p^WM^-CJD cases with mean T2 of 44% (cases 8–11), 5% (cases 12–16), and 0% (cases 17–21), disease duration was 10, 6, and 4 months, respectively (*r* = 0.34) (Table 1). Percent of T2 did not correlate with severity of PrP plaque pathology (sPP) (Table 2). In p^GM^-CJD with T2 ranging from 77 to 19%, cerebellar sPP was 1.3 and 1.4, respectively (*r* = − 0.2). In p^WM^-CJD with T2 ranging from 44% (cases 8–11) and 3.5% (cases 12–18), cerebellar sPP was 1.5 and 1.6 (*r* = − 0.31). Similar results were found when plotting sPP against mean percent T1^21^. In p^WM^-CJD cases with low T1^21^ (24%, cases 10, 12, 15, and 18) and high T1^21^ values (58%, remaining cases), sPP was 1.6 in both groups (*r* = − 0.19). Overall, these data suggest that severity of plaque pathology is not governed by the type of PrP^D^.

### Amyloid β and tau pathology

We carried out Aβ and tau immunostaining of the cerebral cortex, including the medial temporal lobe. Prevalence of Aβ pathology was higher in p^GM^-CJD (86%) than in p^WM^-CJD (50%), but this difference did not reach statistical significance (Table S1). Core Aβ plaques, which were found in ~ 50% of p-CJD cases, were often visible on H&E preparations and were positively stained by thioflavin S (Fig. S8a–c and Table S1). We did not find a significant difference in the prevalence of cerebral amyloid angiopathy (CAA) between p^GM^-CJD (29%) and p^WM^-CJD (43%) cases; subpial Aβ deposits were more frequently seen in p^WM^-CJD (43%) than in p^GM^-CJD (17%) (Table S1). Hyperphosphorylated tau and neurofibrillary tangle pathology was common in both p-CJD subtypes, whereas dystrophic neurites were rare (Fig. S8 d, e and Table S1).

### Clinical features of p-CJD subtypes

The clinical features of p-CJD subtypes largely resembled those of sCJDMM1 and -MM1-2 (Table 3). There were no differences in clinical features between p-CJD subtypes and sCJD when matched by age, gender and type of resPrP^D^; however, statistical comparisons were limited by small sample sizes. Despite the lack of major differences in clinical symptom presentation between the groups, p-CJD mostly presented with cognitive symptoms (72%). Psychiatric presentations were more common in p-CJD compared to sCJD cases (22% vs. 0%), and cerebellar signs were more common in sCJD cases (24% vs. 5.5%). In each of the p-CJD subtypes, CSF 14–3-3 positivity rates were 50%, compared to 94% positivity rate found in sCJD (*P* = 0.03). Other diagnostic tests (total tau, positive RT-QuIC, brain MRI, and EEG with PSWCs), demonstrated similar findings suggestive of prion disease across groups (Table 3). Of cases with a brain MRI suggestive of prion disease, most p-CJD cases demonstrated DWI hyperintensity only in cortical regions (7/8) compared to sCJD control cases, in which 50% (5/10) of cases demonstrated DWI abnormalities in the cortex and caudate nuclei. There were no differences in the clinical phenotype between p-CJD groups.

**Table 3:** Demographic, clinical presentation, biomarkers, and imaging data of US p-CJD and sCJD-129MM controls

Prion disease	p^GM^-CJD (*n* = 7)	p^WM^-CJD (*n* = 14)	sCJD (*n* = 21)^a^	Significance	Statistical test
Female	57^b^ (4/7)^c^	21 (3/14)	38 (8/21)	NS	Fisher’s exact test
Race/Ethnicity					
White	100 (7/7)	92 (12/13)	90 (17/19)		Chi-square
Asian	0 (0/7)	8 (1/13)	5 (1/19)		↓
Other	0 (0/7)	0 (0/13)	5 (1/19)		Fisher’s exact test
Age at onset (years)^d^	67 ± 9	61.5 ± 10.5	64 ± 10		One way ANOVA
Disease duration (months)^d^	7 ± 8	7 ± 4	6 ± 6		↓
Presentation^e^					
Cognitive	67 (4/6)	75 (9/12)	53 (9/17)		Fisher’s exact test
Cerebellar	0 (0/6)	8 (1/12)	24 (4/17)		↓
Sensory	17 (1/6)	8 (1/12)	6 (1/17)		↓
Psychiatric	33 (2/6)	17 (2/12)	0 (0/17)		↓
Other^f^	0 (0/6)	17 (2/12)	12 (2/17)		↓
Family history of prion disease	0 (0/7)	0 (0/9)	0 (0/21)		↓
Family history of neurodegenerative disease	14 (1/7)	0 (0/9)	5 (1/21)		↓
Positive CSF 14–3-3	50 (2/4)	50 (4/8)	94 (15/16)	< 0.03 g	↓
Total tau [pg/ml] × 1000^d^	5.5 ± 3.1 (*n* = 4)	3.1 ± 3.8 (*n* = 7)	5.8 ± 3.7 (*n* = 13)	NS	One way ANOVA
Positive RT-QuIC	80 (4/5)	100 (5/5)	86 (6/7)		Fisher’s exact test
Brain MRI suggestive of CJD	100 (3/3)	100 (6/6)	88 (14/16)		↓
EEG with PSWCs	0 (0/2)	50 (3/6)	25 (3/12)		↓

^a^sCJD−129MM cases were matched for age, gender, and type of PrP^D^

^b^Expressed in percent

^c^Cases with the feature listed/total cases examined

^d^Expressed as mean ± SD

^e^Some cases had more than one type of symptom at clinical presentation

^f^Other symptoms include insomnia, extrapyramidal, visual, and pyramidal

^g^It compares sCJD and p^WM^−CJD

*CSF* cerebrospinal fluid, *RT-QuIC* real−time quaking−induced conversion, *MRI* magnetic resonance imaging, *EEG* electroencephalogram, *PSWC* periodic short−wave complexes; downward arrows: as previous test

### Known risk factors for iatrogenic disease in United States p-CJD patients

Our p^WM^-CJD and p^GM^-CJD case cohorts had a mean age at onset of ~ 62 and 67 years, respectively, and disease duration of ~ 7 months. 76% of p-CJD cases harbored T1-2 and had mean age at onset and disease duration of 65 years and 8 months, respectively, similar to sCJDMM1-2 (62 years and 8 months) [8]. The remaining 24% of p-CJD cases harbored T1 and had a slightly younger age (60 years) and shorter disease duration (6 months) (*P* > 0.05). Only five p^WM^-CJD cases had disease onset prior to 55 years of age, whereas the youngest p^GM^-CJD case was 57 years-old (44–54 years; Table 1). A small subset of four p-CJD cases were hunters and consumed venison (cases 1 and 13–15), but none of them were < 55 years. Only one case (case 11; 52 year-old) had several shunt revisions during their life (Table S2). Comparative assessment of United States (US) p-CJD and age- and-gender matched sCJD cases did not reveal significant differences in recognized acquired prion disease risk factors. Only one patient (case 11) had a history of recognized acquired prion disease risk factors (e.g., neurosurgery) (Tables 1, S2 and S3).

### Transmission study of p^WM^-CJD to mice expressing wild-type and mutated HuPrP

**Fig. 8:**
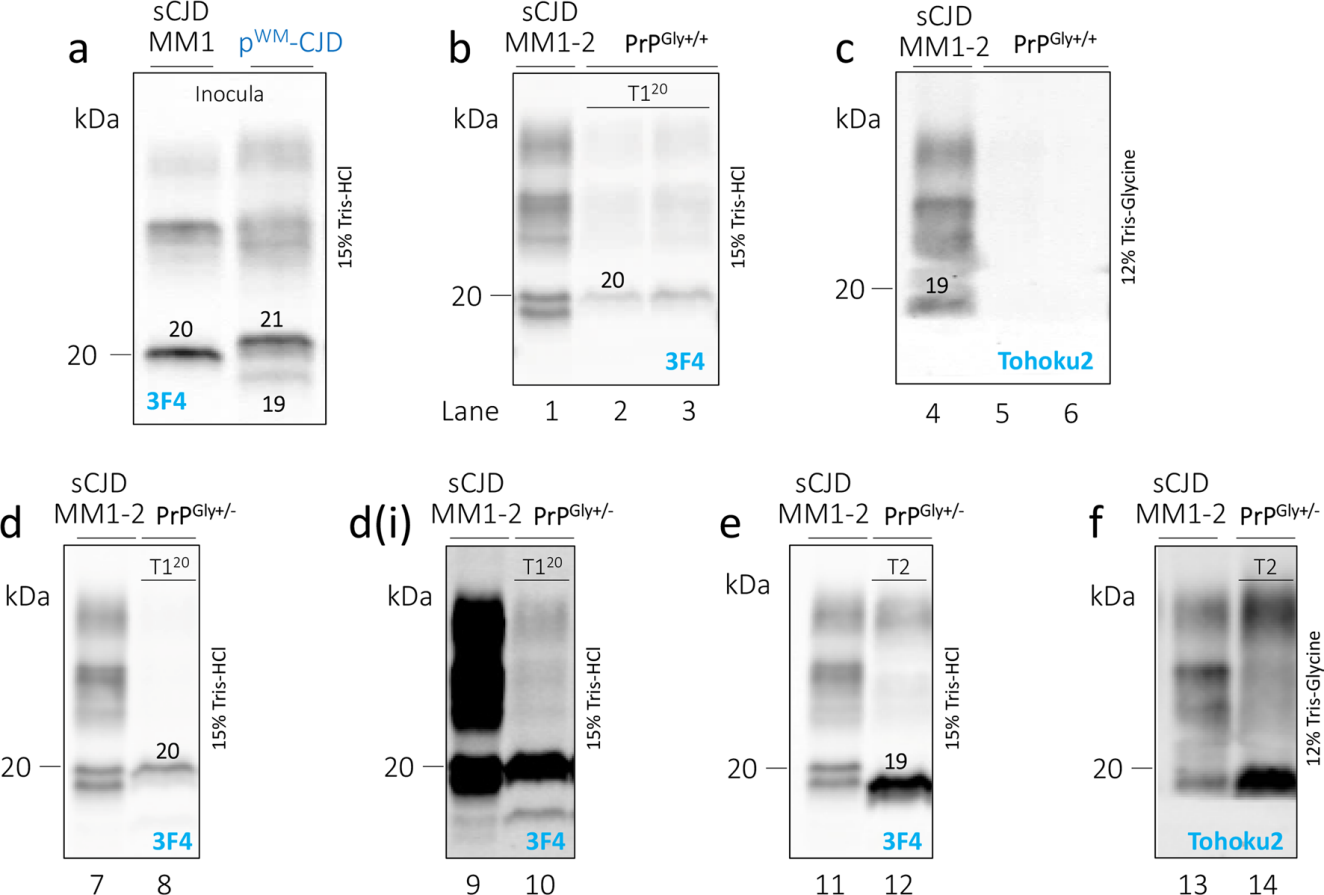
PrP^D^ WB profile of the inocula and diseased mice.** a**–**f**: Brain homogenates (S1) were generated with LB100 pH 8.0, digested with 10 U/ml PK, and probed with 3F4 and tohoku-2 antibodies. We used fully-glycosylated, TgHuPrP^Gly+/+^ (PrP^Gly+/+^), and partially glycosylated, TgHuPrP^Gly+/–^ (PrP^Gly+/–^) mice. PrP bands were resolved by precast 8.7 cm-long gels (LI-COR). Lanes 1, 4, 7, 9, 11, and 13: sCJDMM1-2 control. **a** sCJDMM1 and p^WM^-CJD inocula were obtained from the putamen. Unglycosylated resPrP^D^ T1^20^ of sCJDMM1 (left), and T1^21−20^-T2 of p^WM^-CJD (right). **b** TgHuPrP^Gly+/+^ mice inoculated p^WM^-CJD T1^21−20^-T2 (lane 2) or sCJDMM1 (lane 3) generated T1^20^. **c**: T1^20^ of mice shown in **b** is not recognized by tohoku-2. **d** TgHuPrP^Gly+/–^ mice inoculated sCJDMM1 generated T1^20^. **d**(**i**): Longer signal intensity of **d**, **e**: TgHuPrP^Gly+/–^ mice inoculated p^WM^-CJD generated T2. **f**: T2 of the mouse shown in **e** is detected by tohoku-2. **d**–**f** As expected, resPrP^D^ of TgHuPrP^Gly+/–^-inoculated mice are characterized by the overrepresentation of the unglycosylated isoform. Numbers atop unglycosylated resPrP^D^ bands indicate the relative molecular weight

**Fig. 9:**
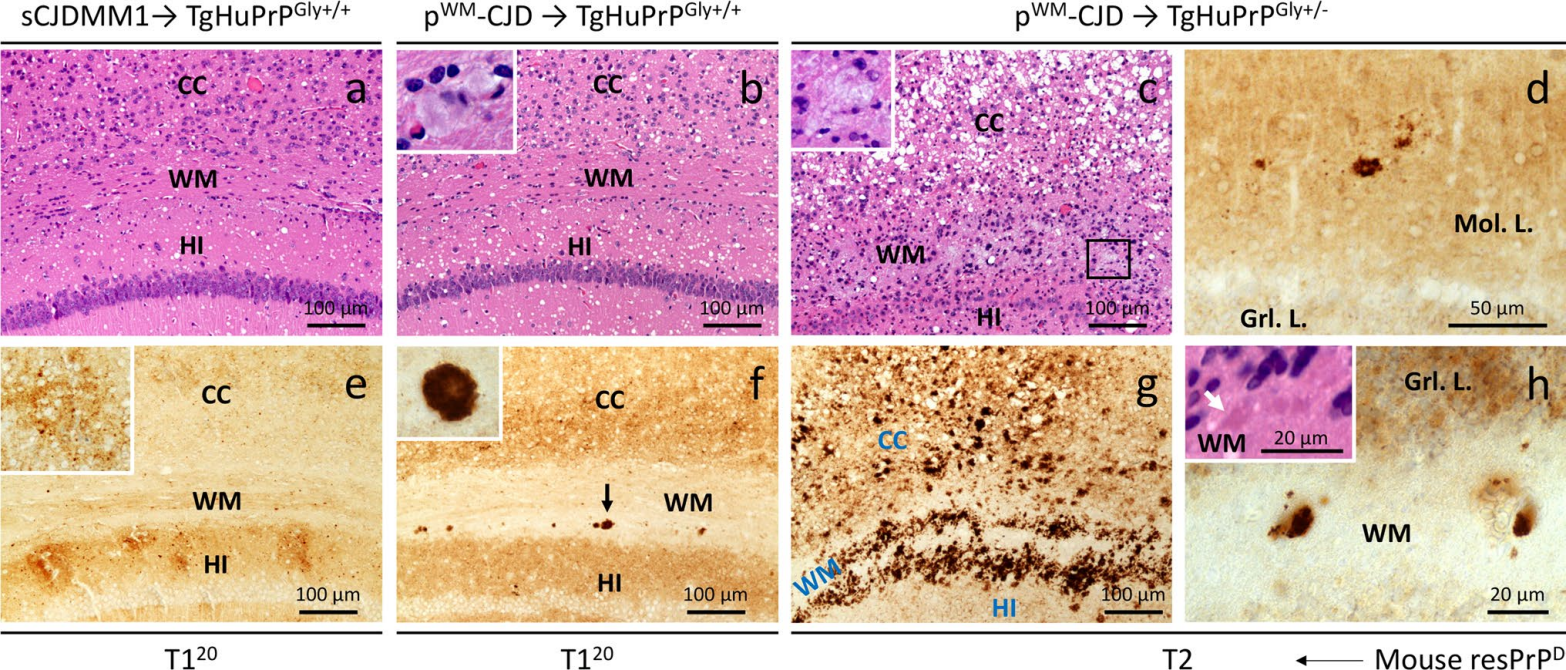
Histopathology of partially and fully glycosylated Tg mice inoculated with p^WM^-CJD and sCJDMM1. Hematoxylin–eosin (H&E) staining (**a**–**c**) and PrP immunohistochemistry (**d**–**h**). **a**-**c**, **e–g**: Hippocampus (HI) and overlaying cerebral cortex (CC). **d**, **h** Cerebellum. **a**, **b** Fine spongiform degeneration (SD) (**a**, **b**) and discrete plaques in white matter (WM) (**b**); inset (**b**): a plaque.** c** Large and confluent vacuoles affecting CC, and amorphous plaque deposits in white matter; inset: amorphous plaques. **e**, **f** Diffuse PrP (**e**, **f**) and PrP plaques (**f**, arrow) affecting the white matter; inset, **e**: higher magnification of diffuse PrP; inset, **f** a PrP plaque. **g** Coarse and perivacuolar PrP deposits in CC and at edges of amorphous plaques. **d**, **h** Plaque-like PrP deposits; inset in **h**: HE showing immature plaques. *Mol. L.* molecular layer, *Grl. L.* granular layer, *antibody* 3F4

We used two Tg mouse lines: (1) Tg mice expressing the WT human PrP-129MM (TgHuPrP^Gly+/+^), and (2) heterozygous Tg mice carrying point mutation (N181Q, N197Q) that eliminated both N-linked glycosylation sites (TgHuPrP^Gly+/–^) (Figs. S2 and S3). Because of the presence of WT HuPrP in the normal allele, and the point mutations on the mutated allele, the western blot profile of PrP^C^ and total PrP was characterized by the predominance of the non-glycosylated PrP isoform in TgHuPrP^Gly+/–^ mice (Figs. S2 and S3). Mice were inoculated with brain homogenates obtained from the putamen of p^WM^-CJD (case 12) and a sCJDMM1 control. resPrP^D^ of p^WM^-CJD was T1^21−20^-T2 (i.e., T1^21−20^ co-existing with T2), whereas that of sCJDMM1 was resPrP^D^ T1^20^ (Fig. 8a). TgHuPrP^Gly+/+^ mice inoculated with p^WM^-CJD and sCJDMM1 become symptomatic after 233 ± 6 and 183 ± 3 days post-inoculation (dpi), respectively (*P* < 0.01) (Table S4). Both groups of mice generated T1^20^ with a PrP glycotype typical of TgHuPrP^Gly+/+^ (Fig. 8b). T1^20^ of TgHuPrP^Gly+/+^ was not detected by the tohoku-2 antibody (Fig. 8c). Histopathology of sCJDMM1-inoculated animals exhibited fine SD throughout the brain (cerebellum was not affected), and diffuse PrP co-distributing with SD (Fig. 9a, e). Mice challenged with p^WM^-CJD showed fine SD, gliosis, and white matter eosinophilic plaques distributed at the border between the hippocampal alveus and the corpus callosum (Fig. 9b). PrP immunostaining showed diffuse PrP in the cerebral cortex and subcortical regions, and discrete PrP granules in the thalamus and lower brain stem. PrP plaques seen on H&E were labeled by the 3F4 antibody (Fig. 9f). The cerebellum was unstained.

Following inoculation with sCJDMM1, TgHuPrP^Gly+/–^ became symptomatic at 272 ± 38 dpi, which is ~ 90 days longer than in the TgHuPrP^Gly+/+^ model (*P* = 0.052) (Table S4). The western blot profile of sCJDMM1-inoculated TgHuPrP^Gly+/–^ mice showed an overrepresentation of the unglycosylated resPrP^D^ T1^20^ isoform (Fig. 8d). Because these mice were found dead, their pathology is not available. Finally, TgHuPrP^Gly+/–^ mice inoculated with p^WM^-CJD showed signs of prion disease at 235 ± 37 dpi, and harbored T2 with a marked representation of the unglycosylated resPrP^D^ isoform (Fig. 8 e, f, and Table S4). These mice showed SD with large vacuoles (Fig. 9c). PrP plaques were often seen in clusters and were noted as (1) discrete entities affecting white and gray matter, or (2) conglomerates of amorphous plaque formations occupying the border between the alveus and the corpus callosum and, perhaps, the deepest layer of the cerebral cortex (Figs. 9c and S8a–d). Immature plaques were noted in the cerebellar white matter (Fig. 9h). Coarse and perivacuolar PrP affected the cerebral cortex, including the hippocampus, while foci of coarse PrP were seen in the subcortical regions (Fig. 9g and data not shown). Notably, small clusters of unicentric plaques were positively labeled by 3F4, while the conglomerates of amorphous plaque-like formations were only stained at the periphery (Figs. 9g and S8 e). As in p^WM^-CJD, rare PrP plaques were noted at the cerebral cortex layer VI (Fig. S9 f). In the cerebellum, rare coarse PrP deposits and plaque-like PrP formations affected the molecular layer and white matter, respectively (Fig. 9 d).

## Discussion

### Prevalence of United States p-CJD

We have characterized the clinico-histopathological and molecular features of 21 US p-CJD cases. Taking into account the 14 p-CJD individuals of the retrospective study, the prevalence of each p-CJD subtype is 1.13% among definite sCJDMM cases, or 0.59% among all sporadic prion diseases. The 0.59% prevalence is higher than the 0.17% of iCJD (*P* = 0.18), but lower than sporadic fatal insomnia (sFI) (1.35%, *P* = 0.059), sCJDVV1 (1.52%, *P* = 0.027), and variably protease-sensitive prionopathy (VPSPr) (1.78%, *P* = 0.0078) [23, 46, 54, 56]. The identification of 7 additional p^WM^-CJD cases suggests that this subtype is the most common of the two. Furthermore, the p^WM^-CJD subtype has been previously described by European and Japanese laboratories [4, 26, 33, 63], whereas a gap exists between our description of p^GM^-CJD and the lack of similar reports in the context of idiopathic CJD [35].

Our study indicates that p^GM^-CJD should be searched in cases of the -MM2 and -MM1-2 sCJD subtypes. On the contrary, 10 p^WM^-CJD cases (71%) belonged to the -MM1 subtype, and only two were diagnosed as -MM2 or -MM1-2 [8]. These prevalences are consistent with those of non-US p^WM^-CJD cases [4, 26, 33, 63].

### Clinical and histopathological features of US- and non-US p^WM^-CJD patients

Data from our case series suggests that there may be slight clinical differences between p-CJD and conventional sCJDMM cases. p-CJD cases may present more commonly with cognitive and psychiatric symptoms and less commonly with cerebellar symptoms compared to sCJDMM cases. Additionally, positive CSF 14–3-3 analyses were less frequent in p-CJD cases, and these cases may be less likely to have basal ganglia involvement on brain MRI. These slight differences fall within the expected clinical heterogeneity of CJD and did not appear to affect the clinical diagnoses of these subjects [1]. Interpretation of the clinical phenotype of p-CJD is limited by the amount and type of clinical information that is collected by the NPDPSC. Although clinical phenotypes can sometimes vary across different human prion strains, they are unlikely to be a reliable indicator of strain differences in isolation. Examples are the prominent neuropsychiatric symptoms observed in both vCJD and young onset sCJD and MRI findings suggestive of sCJD in a case of vCJD that was heterozygous at codon 129 [2, 47]. Clinical features have been shown to be heterogeneous in non-US p^WM^-CJD cases [63]. While age at onset of US and non-US (~ 65 years) p^WM^-CJD cases does not differ significantly, disease duration was 3-times longer in non-US cases (21 ± 12 months; *P* < 0.02) [4, 26, 33, 63]. The significantly different disease duration is probably due to more extensive medical care in Asia.

The US p^WM^-CJD histotype mimicked that of non-US p^WM^-CJD cases [4, 26, 33, 63]. We and others have recently shown that CAA is a major feature of Aβ pathology in patients with iCJD, but not of age-matched sCJD cases [10, 28, 32, 59]. Although the US p-CJD cases were significantly older than US sCJDMM cases [10], CAA prevalence did not differ between the two diseases. These results point to an age-dependent Aβ pathology in US p-CJD.

### US p^GM^-CJD, sporadic CJD and iCJD cases with PrP plaque pathology: a review of the literature

PrP plaques populated the cerebellar cortex in p^GM^-CJD with the exception of one case in which rare PrP plaques affected the occipital cortex. The spread of PrP plaques to the occipital lobe does not seem to be the result of a protracted disease duration since death occurred two months after clinical presentation. Two p^GM^-CJD cases showed target-like PrP formations. Whether these “loose plaques” contains PrP fibrils remains to be determined [67]. Also, the presence of only rare diffuse Aβ plaques in these patients is against the hypothesis that target-like PrP is the result of an enhancement of PrP around or within Aβ plaques [10].

We have searched in the literature for the presence of PrP plaques in the gray matter of patients with sporadic prion disease linked to codon 129MM genotype; three cases were found. In the first report, a 54-year-old neurosurgeon with an 18-month disease course and sleep disturbances harbored PrP plaques in the cerebral cortex. Inoculation of brain homogenates from this patient to chimpanzee and squirrel monkeys lead to prion disease. However, the human histotype was not fully reproduced by these primates as kuru-type plaques were not detected in the brain of the affected animals [64]. In another study, a 75-year-old woman with an 11-month clinical course, underwent neurosurgery without dura mater about 14 years before the onset of clinical symptoms. At autopsy, “congophilic amyloid plaques” were noted in the cerebellar cortex [31]. Although both patients were originally diagnosed as being sporadic, a subsequent study suggested these two cases had an iatrogenic prion disease. This conclusion, which stems from a known iatrogenic risk factor in one of the cases (neurosurgery), was supported by an in vivo study [35]. The third case is that of a 40-year-old woman with no known history of iatrogenic exposure who was alive at the time of the brain biopsy, which occurred ~ 2.5 years after the appearance of clinical disease. This patient presented with dementia, showed a “conspicuous” number of PrP plaques in the occipital cortex, and was 129MM [45]. To our knowledge, these cases were free of florid plaques.

In our p^GM^-CJD case 5, clusters of florid plaques were noted in the cerebellum. Our p^GM^-CJD case 5, is a 57-year-old male with sensory symptoms (numbness, tingling, and pain in the fingers) at presentation. Case 5 did not have a history of venison consumption, blood transfusion, travel to any BSE-exposed countries, or known surgical history. It should be emphasized that disease onset with sensory symptoms can be a clinical presentation observed in vCJD; however, case 5’s brain MRI demonstrated restricted diffusion abnormalities in the cortex and caudate that is typical for sCJD. Additionally, the illness duration of 2 months is shorter than typically reported in vCJD. The resPrP^D^ glycotype of case 5 resembles that of sCJD.

The presence of florid plaques has been described in a 70-year-old Slovenian female who presented with psychiatric symptoms at the age of ~ 68 years, and had traveled to the UK at a time of BSE pandemic. The patient was of the 129MV genotype and harbored resPrP^D^ T2. Despite the presence of florid plaques and several clinical features overlapping with vCJD, the authors concluded that the patient’s atypical phenotype was likely due to the known heterogeneity of the -MV2 subtype [6, 49, 57]. The resPrP^D^ glycotype of this patient mimicked that of sCJD, characterized by the predominance of the monoglycosylated resPrP^D^ isoform. By contrast, the over-representation of the diglycosylated resPrP^D^ isoform is a feature of vCJD, and is independent of the codon 129 genotype [47, 71]. Florid plaques have been reported in patients with iCJD linked to the 129MM genotype (iCJDMM) [13, 40, 42, 66]. We have described a US growth hormone iCJDMM (GH-iCJDMM) case with a complex PrP plaque pathology. In addition, this patient showed laminar spongiform degeneration, PrP immunostaining with diffuse, plaque-like and perineuronal patterns, and pericellular PrP [13]. Thus, the presence of PrP plaques is the only common histopathological feature of the US GH-iCJDMM and p^GM^-CJD. Moreover, PrP plaque pathology of the US GH-iCJDMM case was significantly more severe.

Finally, conflicted results are shown in the literature regarding the gel mobility of the unglycosylated resPrP^D^ of iCJDMM cases with PrP plaques. In one case report, resPrP^D^ of DM-iCJD migrated about 1 kDa more than the ~ 21 kDa of sCJDMM1 [42]. Similar results were observed in one US GH-iCJD [10, 13], two atypical iCJD [35], Japanese DM-iCJD [34], and one UK GH-iCJD case[60]. In two other studies, resPrP^D^ of iCJD and sCJDMM1 showed similar gel mobility [20, 66]. Notably, 10 of 11 French iCJDMM cases harbored “Type 1” and only one case “type i” (or type “intermediate”, corresponding to a resPrP^D^ fragment of ~ 20 kDa) [20]. Since the buffer pH and the use of other stringent experimental conditions are important tools in assessing the gel mobility of resPrP^D^, unified experimental conditions should be used to characterize the molecular features of atypical cases that are suspected of an iatrogenic etiology (Fig. S10).

### Molecular features of T1 and T2 of US p-CJD

The electrophoretic profile of resPrP^D^ T1 and its anatomical distribution are major differences of p-CJD subtypes. Although T1^21−20^ was noted in ~ 55% of the cases, its prevalence is likely to be higher if a more extensive sampling of subcortical brain regions is carried out. T1^21−20^ was occasionally detected in the cerebral cortex, but never found in the cerebellum. These data suggest a tropism of PrP^D^ for different neuronal cell types, and high accessibility of T1^21^ to subcortical regions, where PrP^C^ is likely to be converted to PrP^D^ T1^21^ at a higher rate than T1^20^. This hypothesis is supported by the fact that our buffer pH (8.0) favors the formation of T1^20^ [8, 9, 50]. Furthermore, it seems unlikely that T2 affects T1 distribution in subcortical regions, as the amount of T2 distribution in the two p-CJD subtypes was virtually identical. The fact that T1^20^ of p^GM^-CJD and sCJDMM1 share similar GdnHCl_1/2_ values based on the CSSA, does not necessarily indicate that T1^20^ in these two diseases belong to the same prion strain. This can be demonstrated by the similar GdnHCl_1/2_ indexes of T1^20^ and T2 associated with sCJDVV1 and -VV2, respectively [14]. In previous studies, RT-QuIC assays have revealed some significant differences in the means of kinetic values obtained from certain sCJD subtypes [21]. In this study, we saw modest differences in the overall mean times to threshold between p^WM^-CJD T1 and p^GM^-CJD T2 brain specimens, but the relevance of such differences is unclear because they could be explained by relative seed concentration and distinct seed structures. A second major molecular difference, is the higher proportion of T2 and sCJDMM2-like histopathological features in p^GM^-CJD, which exceeded that of p^WM^-CJD by ~ 3- and twofold, respectively. Despite the significantly higher proportion of T2 in p^GM^-CJD, T1^20^ is better represented than T2 overall, and the bulk of PrP plaque pathology is in the cerebellum, which harbors only T1^20^ by western blot in most cases. Case 7, with an overall T2 representation of only 3%, deserves a separate analysis. Unlike the other p^GM^-CJD cases, rare PrP plaques populated only the granular layer. This may indicate that the presence of T2 is required for a more severe and/or widespread distribution of PrP plaques, and that T2 aggregates are preferentially sequestered in the amyloid core [68]. Furthermore, cerebellar T1 aggregates may be less compact and readily disaggregate following proteolytic digestion and standard denaturation procedures [37].

### Do T1^21^, T1^20^ and T2 of p^WM^-CJD have strains properties?

Transmission studies are a gold standard to gain insights on disease mechanism of neurodegeneration. From our bioassay we can reach three conclusions. First, only p^WM^-CJD T1^20^ propagates in TgHuPrP^Gly+/+^, generating amyloid plaques. Although we did not perform a second passage, T1^20^ of p^WM^-CJD and sCJDMM1 likely represent different prion strains. The lack of T2 in these mice indicates that T1^20^ is a faster replicating prion strain [8, 52] (Table 1). Second, T1^21^ could be a different human strain, as it did not propagate in the host. One possible, but perhaps unlikely, explanation is that T1^21^ in the cerebral cortex (where the brain homogenates was injected), may be unable to convert PrP^C^ into PrP^D^; or that conversion by T1^21^ is inefficient and occurs at a rate that is several orders of magnitudes lower than that of T1^20^. Recently, we have shown that T1^21^ associated with sCJDVV1 faithfully propagates in the TgHuPrP-129VV mouse brain and that inoculation of T1^21−20^ to the same mouse line generates T1^21^ [11]. However, attempts to transmit T1^21^ to TgHuPrP-129MM mice failed on first and second passages. Altogether, the present data and those of Cali et al. [11] suggest that T1^21^ of p^WM^-CJD and sCJDVV1 have distinct molecular features, as p^WM^-CJD T1^21^ does not replicate in TgHuPrP-129MM mice, whereas sCJDVV1 T1^21^ faithfully propagates to TgHuPrP mice with the same genotype (129VV). It would be important to assess whether T1^21^ p^WM^-CJD faithfully transmits disease to TgHuPrP-129VV mice. Lastly, p^WM^-CJD injected into TgHuPrP^Gly+/–^ mice generated T2 and PrP plaques. These data suggest that the lack of glycosylated PrP isoforms, even in one allele, is sufficient to favor T2 propagation, and therefore overturn strain selection by the host. The incomplete glycosylation may also explain the more complex PrP plaque pathology. Although the formation of PrP plaques in TgHuPrP^Gly+/–^ mice may be the result of the partial glycosylation, the presence of immature plaques in the cerebellar white matter argues for a role of T2 (present in the inoculum) in PrP plaque formation. A minority of TgHuPrP^Gly+/–^ mice were accessible to T1^20^ of sCJDMM1 but the incubation time was longer than in TgHuPrP^Gly+/+^ mice. Overall, our data suggest that glycans play a protective role in these mice [65, 73]. Although the partial absence of glycans accelerates T2 replication at the expenses of T1^20^, it should be emphasized that T1 in sCJDMM patients is significantly better represented than T2.

## Conclusions

We have characterized the clinical, histopathological, and molecular properties of two human prion diseases with distinct PrP plaque pathology and divergent PrP^D^ molecular features. While p^WM^-CJD cases are readily identifiable due to the presence of PrP plaques on a white background, p^GM^-CJD cases may be more difficult to detect. If p^GM^-CJD is a sporadic prion disease, this phenotype should be identified by other prion Surveillance Centers. Nevertheless, the lack of major reports on p^GM^-CJD by other countries is puzzling as both plaque histotypes have similar prevalences in the United States. If these cases are the result of acquired prion disease, their route of transmission is not apparent and not due to any recognized or currently hypothesized acquired prion disease/risk factors. While one major goal of this study is to contribute to the identification of atypical or novel histotypes (or novel prion diseases), it is important to identify new markers of iatrogenic disease. Meanwhile, it seems appropriate to classify p^GM^-CJD and p^WM^-CJD as sporadic prion diseases. Additionally, in vitro and in vivo experiments are needed to further dissect the molecular features of p-CJD PrP^D^ with the aim of gaining insights on the mechanisms governing these disorders.

## Supplementary Information

Below is the link to the electronic supplementary material.

Supplementary file1 (DOCX 17663 KB)

Supplementary file2 (DOCX 26 KB)

Supplementary file3 (DOCX 31 KB)

Supplementary file4 (DOCX 25 KB)

Supplementary file5 (DOCX 26 KB)

## Data Availability

The data supporting the findings of this study are included in tables and supplemental materials.

## Abbreviations

CJD: Creutzfeldt–Jakob disease
sCJD: Sporadic CJD
iCJD: Iatrogenic CJD
p-CJD: Sporadic CJD cases with PrP plaque pathology
p^GM^-CJD: sCJD with PrP plaques affecting the gray matter
p^WM^-CJD: sCJD with PrP plaques affecting the white matter
GH: Growth hormone
DM: Dura mater
sCJDMM: Sporadic sCJD harboring codon 129MM genotype
vCJD: Variant CJD
BSE: Bovine spongiform encephalopathy
CWD: Chronic wasting disease
WB: Western blot
total PrP: PrP^C^ + PrP^D^
T1: Type 1
T2: Type 2
T1-2: Co-occurrence of PrP^D^ types 1 and 2
PrP: Prion protein
PrP^C^: Cellular PrP
PrP^D^: Disease-associated PrP
PK: Proteinase K
resPrP^D^: PK-resistant PrP^D^
T1^21^ and T1^20^: T1 variants with unglycosylated resPrP^D^ isoform of ~ 21 and ~ 20 kDa, respectively
T1^21^^−20^: T1 variant with unglycosylated resPrP^D^ doublet of ~ 21–20 kDa characterized by a prominent 21 kDa band
M: Methionine
V: Valine
CSSA: Conformational solubility and stability assay
RT-QuIC: Real-time quaking-induced conversion
sPP: Severity of PrP plaque pathology
Aβ: Amyloid β
TgHuPrP^Gly+/+^: Transgenic mice (Tg) expressing wild-type human PrP
TgHuPrP^Gly+/^: Tg mice expressing the wild-type and mutated human PrP
BH: Brain homogenate
H&E.: Hematoxylin–eosin staining
SD: Spongiform degeneration
Dpi: Days post-inoculation
