# Correction: A novel subtype of sporadic Creutzfeldt–Jakob disease with PRNP codon 129MM genotype and PrP plaques

**DOI:** 10.1007/s00401-023-02592-y

**Published:** 2023-05-30

**Authors:** Rabeah Bayazid, Christina Orru’, Rabail Aslam, Yvonne Cohen, Amelia Silva-Rohwer, Seong-Ki Lee, Rossana Occhipinti, Qingzhong Kong, Shashirekha Shetty, Mark L. Cohen, Byron Caughey, Lawrence B. Schonberger, Brian S. Appleby, Ignazio Cali

**Affiliations:** 1 Department of Pathology, School of Medicine, Case Western Reserve University, Cleveland, OH USA; 2 Department of Physiology and Biophysics, School of Medicine, Case Western Reserve University, Cleveland, OH USA; 3 Department of Neurology, School of Medicine, Case Western Reserve University, Cleveland, OH USA; 4 Department of Psychiatry, School of Medicine, Case Western Reserve University, Cleveland, OH USA; 5 National Prion Disease Pathology Surveillance Center, Cleveland, OH USA; 6 Laboratory of Persistent Viral Diseases, NIH, Hamilton, MT USA; 7 Division of High-Consequence Pathogens and Pathology, National Center for Emerging and Zoonotic Infectious Diseases, Centers for Disease Control and Prevention, Atlanta, GA USA

**Correction: Acta Neuropathologica** 10.1007/s00401-023-02581-1

In the original publication, Tables 1 to 3 were incorrectly formatted during production process.

The correct format of Tables 1, 2 and 3 are given below.

**Table 1:** Case-cohort of US CJD with PrP plaques (p) affecting the grey matter (p^GM^-CJD) and white matter (p^WM^-CJD)

Case number	Year case ID	PK-resistant PrP^D^			Gender, female (%)	Race, white (%)	Age at onset (years)	Disease duration (months)	Cases found by retrospective study^b^	Available frozen tissue subcortical regions
		Type 1–2 (%)	T2^a^ (%)							
			Brain	Crbl						
p^GM^-CJD										
1	2015	1–2	98	ND	−	+	63	12	Yes	Yes
2	2016	1–2	89	100	+	+	73	4	Yes	Yes
3	2015	1–2	45	10	−	+	68	24	Yes	Yes
4	2016	1–2	29	0	+	+	71	2	Yes	Yes
5	2015	1–2	26	0	−	+	57	2	Yes	Yes
6	2016	1–2	16	0	+	+	60	2	Yes	Yes
7	2014	1–2	3	0	+	+	83	2	Yes	Yes
	2015–16	100	44 ± 36^e^	18 ± 40^e^	57	100	67 ± 9^e^	7 ± 8^e^	100%	100%
p^WM^-CJD										
8	2017	1–2	60	ND	+	+	72	9	Yes	Yes
9^c^	2005	1–2	50	0	−	+	54	11	No	Yes
10^d^	2018	1–2	40	0	−	−	69	7	No	Yes
11^c^	2003	1–2	24	0	−	+	52	11	No	Yes
12^c^	2006	1–2	8	0	−	+	59	7	No	Yes
13	2015	1–2	5	ND	−	+	75	1	Yes	Yes
14^d^	2019	1–2	5	0	−	+	51	7	No	Yes
15	2016	1–2	4	0	+	+	72	9	Yes	Yes
16^c^	2010	1–2	2	0	−	+	57	13	No	Yes
17	2015	1	0	0	−	+	62	7	Yes	Yes
18	2016	1	0	0	−	+	44	3	Yes	Yes
19^c^	2011	1	0	0	−	U	76	5	No	No
20	2013	1	0	NA	−	+	68	2	Yes	No
21	2016	1	0	0	+	+	50	11	Yes	No
	2003–19	64	14 ± 21^e,f^	0	21	92	61.5 ± 10.5^e^	7 ± 4^e^	50%	79%

No statistical differences were found when comparing gender, race, age at onset, and disease duration

*ID* identification, *ND* not detected, *NA* not available, *U* unknown.

^a^Percentage of PK-resistant PrP^D^ (resPrP^D^) T2 out of total resPrP^D^ (i.e., total resPrP^D^ = resPrP^D^ T1 + resPrP^D^ T2) averaged from 5 brain regions (cases 1–18), two regions (cases 19 and 21) or available in one brain region (case 20)

^b^620 sCJD cases examined

^c, d^Cases identified ^b^before or ^c^after retrospective histopathological examination.

^e^Expressed as mean ± SD

^f^P < 0.03 (Student’s t test)

**Table 2:** Molecular and histopathological features of US p^GM^- and p^WM^-CJD subtypes

Case number	Age at onset (years)	Disease duration (months)			PrP immunostaining pattern							
					MM2-like CC (%)^d^	Plaques						
						CC^e,f^	Subc^e,f^	Cerebellum				
			PK-resistant PrP^D^					Grey matter		White matter	Coarse	“Brush stroke-like”
			T1^21^ (%)^a^	T2 (%)^b, c^				Mol. L	Grl. L.^g^		Crbl Mol. L	Crbl Mol. L
p^GM^-CJD												
1	63	12	0	98	90	0	0	3	1	0	+	−
2	73	4	0	89	53	0	0	2	0.5	0	+	+
3	68	24	0	45	90	0	0	1	0.5	0	+	+
4	71	2	0	29	20	0	0	1.5	1	0	−	+
5	57	2	0	26	40	0	0	2	1	0	−	+
6	60	2	0	16	43	1	0	3	2	0.1	−	+
7	83	2	0	3	5	0	0	0	1	0.1	−	+
Mean ± SD	68 ± 9	7 ± 8	0	44 ± 36	49 ± 32	0.1 ± 0.4	0	1.8 ± 1.1	1 ± 0.5	0.03 ± 0.05	43^h^ (3/7) ^i^	86^h^ (6/7) ^i^
p^WM^-CJD												
8	72	9	67	60	80	0.5	1.5	0	0	2	+	+
9	54	11	55	50	57	1.5	2	0	0	2	+	−
10	69	7	23	40	37	0.5	2	0	0	2	+	+
11	52	11	65	24	20	1	1.5	0	0	2	−	+
12	59	7	25	8	10	2 ^j^	1.5	0	0	1	+	+
13	75	1	62	5	5	0.5	1	0	0	1	−	+
14	51	7	52	5	0	0.5	1	0	0	1	−	+
15	72	9	27	4	30	0.5	3	0	0	2	+	+
16	57	13	46	2	0	3 ^j^	3	0	0	3	−	+
17	62	7	56	0	0	2	3	0	0	2	−	+
18	44	3	22	0	0	1.5	1.5	0	0	0.5	−	+
Mean ± SD	61 ± 10	8 ± 3.5	45 ± 18	18 ± 22	22 ± 27	1.2 ± 0.8	1.9 ± 0.8	0	0	1.7 ± 0.7	45^h^ (5/11)^i^	91^h^ (10/11)^i^
P value	NS	NS	< 0.0001	NS	NS	< 0.007	< 0.0001	< 0.005	< 0.002	< 0.0001	NS^k^	NS^l^

*Mol. L.* molecular layer, *Crbl* cerebellum, *Grl. L.* granular layer, *NS* not significant

^a^Percentage of PK-resistant PrP^D^ (resPrP^D^) T1^21^ out of total resPrP^D^ T1 (i.e., total T1 = T1^21^ + T1^20^) averaged from subcortical (Subc) regions (putamen and thalamus)

^b,c^Data obtained from the ^b^cerebral cortex (frontal and occipital cortices), and ^c^expressed as percentage of resPrP^D^ type 2 (T2) out of total resPrP^D^ (i.e., total resPrP^D^ = resPrP^D^ T1 + resPrP^D^ T2)

^d^Data expressed as percentage of the surface of cerebral cortex (frontal, temporal and occipital cortices) occupied by coarse and/or perivacuolar IP

^e,f^It refers to ^e^grey and white matter (cases 1–7) or ^f^white matter (cases 8–18)

^g^It includes the Purkinje cell layer

^h^Expressed in %

^i^Cases with the feature listed/total cases examined

^j^Rare PrP plaques affecting layer VI of the cerebral cortex. PrP plaque severity was scored on a 0–3 scale (0, absent; 1, mild; 2, moderate; 3, severe). Statistical significance was calculated using Student’s t test

^k^Fisher’s exact test or ^l^ Chi-square test

**Table 3:** Demographic, clinical presentation, biomarkers, and imaging data of US p-CJD and sCJD-129MM controls

Prion disease	p^GM^-CJD (n = 7)	p^WM^-CJD (n = 14)	sCJD (n = 21)^a^	Significance	Statistical test
Female	57^b^ (4/7)^c^	21 (3/14)	38 (8/21)	NS	Fisher’s exact test
Race/ethnicity					
White	100 (7/7)	92 (12/13)	90 (17/19)		Chi-square
Asian	0 (0/7)	8 (1/13)	5 (1/19)		↓
Other	0 (0/7)	0 (0/13)	5 (1/19)		Fisher’s exact test
Age at onset (years)^d^	67 ± 9	61.5 ± 10.5	64 ± 10		One way ANOVA
Disease duration (months)^d^	7 ± 8	7 ± 4	6 ± 6		↓
Presentation^e^					
Cognitive	67 (4/6)	75 (9/12)	53 (9/17)		Fisher’s exact test
Cerebellar	0 (0/6)	8 (1/12)	24 (4/17)		↓
Sensory	17 (1/6)	8 (1/12)	6 (1/17)		↓
Psychiatric	33 (2/6)	17 (2/12)	0 (0/17)		↓
Other^f^	0 (0/6)	17 (2/12)	12 (2/17)		↓
Family history of prion disease	0 (0/7)	0 (0/9)	0 (0/21)		↓
Family history of neurodegenerative disease	14 (1/7)	0 (0/9)	5 (1/21)		↓
Positive CSF 14-3-3	50 (2/4)	50 (4/8)	94 (15/16)	< 0.03 g	↓
Total tau [pg/ml] × 1000^d^	5.5 ± 3.1 (n = 4)	3.1 ± 3.8 (n = 7)	5.8 ± 3.7 (n = 13)	NS	One way ANOVA
Positive RT-QuIC	80 (4/5)	100 (5/5)	86 (6/7)		Fisher’s exact test
Brain MRI suggestive of CJD	100 (3/3)	100 (6/6)	88 (14/16)		↓
EEG with PSWCs	0 (0/2)	50 (3/6)	25 (3/12)		↓

*CSF* cerebrospinal fluid, *RT-QuIC* real-time quaking-induced conversion. *MRI* magnetic resonance imaging, *EEG* electroencephalogram, *PSWC* periodic short-wave complexes, *downward arrows* as previous test

^a^sCJD-129MM cases were matched for age, gender, and type of PrP^D^

^b^Expressed in percent

^c^Cases with the feature listed/total cases examined

^d^Expressed as mean ± SD

^e^Some cases had more than one type of symptom at clinical presentation

^f^Other symptoms include insomnia, extrapyramidal, visual, and pyramidal

^g^It compares sCJD and p^WM^-CJD

