# Supplementary material for: A novel subtype of sporadic Creutzfeldt–Jakob disease with PRNP codon 129MM genotype and PrP plaques
Source: Acta Neuropathol. 2023 May 8;146(1):121–43. doi: 10.1007/s00401-023-02581-1 (PMC10166463; doi:10.1007/s00401-023-02581-1)

**A novel subtype of sporadic Creutzfeldt-Jakob disease with *PRNP* codon 129MM genotype and PrP plaques**

Rabeah Bayazid,^1^ Christina Orru’,^6^ Rabail Aslam,^1^ Yvonne Cohen,^1^ Amelia Silva-Rohwer,^1,5^ Seong-Ki Lee, ^2^ Occhipinti R.,^2^ Qingzhong Kong,^1,5^ Shashirekha Shetty,^1,5^ Mark L. Cohen,^1,5^ Byron Caughey,^6^ Lawrence B. Schonberger,^7^ Brian S. Appleby,^1,3,4,5^ Ignazio Cali.^1,5^

Departments of ^1^Pathology, ^2^Physiology and Biophysics, ^3^Neurology, and ^4^Psychiatry, School of Medicine, Case Western Reserve University; ^5^National Prion Disease Pathology Surveillance Center, Cleveland, OH 44106, USA; ^6^Laboratory of Persistent Viral Diseases, NIH; ^7^Division of High-Consequence Pathogens and Pathology, National Center for Emerging and Zoonotic Infectious Diseases, Centers for Disease Control and Prevention, Atlanta, GA, USA.

Corresponding author: [ixc20@case.edu](mailto:ixc20@case.edu)

**MATERIALS AND METHODS**

**Reagents and antibodies**

Sodium deoxycholate, sodium chloride (NaCl), Tris-HCl, Nonidet P-40, 10X Dulbecco phosphate buffered saline (DPBS), proteinase K (PK), phenylmethanesulfonyl fluoride (PMSF), Kodak Biomax MR and XAR films and Thioflavin S were purchased from Sigma Aldrich (St. Louis, MO, USA); sodium dodecyl sulfate (SDS), 10X Tris buffered saline (TBS), Tween 20, β-mercaptoethanol, 15% Criterion™ Tris–HCl precast Gels (W x L: 13.3 cm × 8.7 cm), 30% Acrylamide/Bis solution, tetramethylethylenediamine (TEMED), 10% sodium dodecyl sulfate (SDS) and ammonium persulphate (APS) from Bio-Rad Laboratories (Hercules, CA, USA); Odyssey blocking buffer from LI-COR Biosciences (Lincoln, NE, USA); methanol, ethylenediaminetetraacetic acid (EDTA), guanidine hydrochloride (GdnHCl) from Promega (Madison, WI, USA); polyvinylidene difluoride (PVDF) membrane (Immobilon-FL) from EMD Millipore (Billerica, MA, USA). The peptide N-glycosidase F from New England Biolabs Inc. (Beverly, MA, USA). Antibodies used were: 3F4 to human PrP (HuPrP) residues 106-110[7], and tohoku-2 to HuPrP residues 97-103[9]; 12B2 and 1E4 to HuPrP residues 89-93 and 97-108, respectively, were from Wageningen University & Research (Lelystad, Netherlands)[11]; antibodies 4G8, to human Amyloid-β (Aβ) residues 17–24, and AT8, to human phospho-tau residues Ser202 and Thr205, were from Thermo Fisher Scientific Inc. (Waltham, MA, USA).

**REFERENCES**

1. Cali I, Castellani R, Alshekhlee A, Cohen Y, Blevins J, Yuan J, Langeveld JPM, Parchi P, Safar JG, Zou W-Q, Gambetti P (2009) Co-existence of scrapie prion protein types 1 and 2 in sporadic Creutzfeldt-Jakob disease: its effect on the phenotype and prion-type characteristics. Brain 132:2643–2658. doi: 10.1093/brain/awp196

2. Cali I, Castellani R, Yuan J, Al-Shekhlee A, Cohen ML, Xiao X, Moleres FJ, Parchi P, Zou W-Q, Gambetti P (2006) Classification of sporadic Creutzfeldt-Jakob disease revisited. Brain 129:2266–2277. doi: 10.1093/brain/awl224

3. Cali I, Cohen ML, Haїk S, Parchi P, Giaccone G, Collins SJ, Kofskey D, Wang H, McLean CA, Brandel J-P, Privat N, Sazdovitch V, Duyckaerts C, Kitamoto T, Belay ED, Maddox RA, Tagliavini F, Pocchiari M, Leschek E, Appleby BS, Safar JG, Schonberger LB, Gambetti P (2018) Iatrogenic Creutzfeldt-Jakob disease with Amyloid-β pathology: an international study. Acta Neuropathol Commun 6:5. doi: 10.1186/s40478-017-0503-z

4. Cali I, Miller CJ, Parisi JE, Geschwind MD, Gambetti P, Schonberger LB (2015) Distinct pathological phenotypes of Creutzfeldt-Jakob disease in recipients of prion-contaminated growth hormone. Acta Neuropathol Commun 3:37. doi: 10.1186/s40478-015-0214-2

5. Duyckaerts C, Sazdovitch V, Ando K, Seilhean D, Privat N, Yilmaz Z, Peckeu L, Amar E, Comoy E, Maceski A, Lehmann S, Brion J-P, Brandel J-P, Haïk S (2017) Neuropathology of iatrogenic Creutzfeldt-Jakob disease and immunoassay of French cadaver-sourced growth hormone batches suggest possible transmission of tauopathy and long incubation periods for the transmission of Abeta pathology. Acta Neuropathol. doi: 10.1007/s00401-017-1791-x

6. Gelpi E, Soler Insa JM, Parchi P, Saverioni D, Yagüe J, Nos C, Martínez-Saez E, Ribalta T, Ferrer I, Sanchez-Valle R (2013) Atypical neuropathological sCJD-MM phenotype with abundant white matter Kuru-type plaques sparing the cerebellar cortex. Neuropathology 33:204–208. doi: 10.1111/j.1440-1789.2012.01341.x

7. Kascsak RJ, Rubenstein R, Merz PA, Tonna-DeMasi M, Fersko R, Carp RI, Wisniewski HM, Diringer H (1987) Mouse polyclonal and monoclonal antibody to scrapie-associated fibril proteins. J Virol 61:3688–3693

8. Kobayashi A, Matsuura Y, Mohri S, Kitamoto T (2014) Distinct origins of dura mater graft-associated Creutzfeldt-Jakob disease: past and future problems. Acta Neuropathol Commun 2:32. doi: 10.1186/2051-5960-2-32

9. Kobayashi A, Sakuma N, Matsuura Y, Mohri S, Aguzzi A, Kitamoto T (2010) Experimental verification of a traceback phenomenon in prion infection. J Virol 84:3230–3238. doi: 10.1128/JVI.02387-09

10. Kretzschmar HA, Sethi S, Földvári Z, Windl O, Querner V, Zerr I, Poser S (2003) Latrogenic Creutzfeldt-Jakob disease with florid plaques. Brain Pathol 13:245–249. doi: 10.1111/j.1750-3639.2003.tb00025.x

11. Langeveld JPM, Jacobs JG, Erkens JHF, Bossers A, van Zijderveld FG, van Keulen LJM (2006) Rapid and discriminatory diagnosis of scrapie and BSE in retro-pharyngeal lymph nodes of sheep. BMC Vet Res 2:19. doi: 10.1186/1746-6148-2-19

12. Ritchie DL, Barria MA, Peden AH, Yull HM, Kirkpatrick J, Adlard P, Ironside JW, Head MW (2017) UK Iatrogenic Creutzfeldt-Jakob disease: investigating human prion transmission across genotypic barriers using human tissue-based and molecular approaches. Acta Neuropathol 133:579–595. doi: 10.1007/s00401-016-1638-x

13. Rossi M, Saverioni D, Di Bari M, Baiardi S, Lemstra AW, Pirisinu L, Capellari S, Rozemuller A, Nonno R, Parchi P (2017) Atypical Creutzfeldt-Jakob disease with PrP-amyloid plaques in white matter: molecular characterization and transmission to bank voles show the M1 strain signature. Acta Neuropathologica Communications 5:87. doi: 10.1186/s40478-017-0496-7

**FIGURE LEGENDS**

**Fig. S1** Gel mobility of p^GM^-CJD and sCJD resPrP^D^ following tissue homogenized with LB100 pH 6.9. Brain homogenates (S1) were digested with 100 U/ml PK (~ 1 mg/ml), probed with 3F4, and developed with a chemiluminescent substrate. The WB profile of the unglycosylated isoform of resPrP^D^ T1 appears as a major fragment of ~ 21 kDa in p^GM^-CJD and sCJDMM1. In sCJDMV2K and -MM2, resPrP^D^ is characterized by a ~ 20-19 kDa doublet and ~ 19 kDa band, respectively. Numbers atop each unglycosylated resPrP^D^ band indicate the relative molecular weight.

**Fig. S2** Schematic representation of wild-type and mutated PrP^C^ and WB profiles of total PrP in p^WM^-CJD-inoculated mice. **a.** Left: Each PrP^C^ isoform is represented by the black bar; red circles refer to glycans linked to asparagine (N) 181 (N181) and N197 residues; (#) either N181 or N197 residue could be glycosylated. Center: Cartoon depicting PrP^C^ glycoforms as they appear on western blot. Mice carrying PrP^C^ Gly^+/-^ harbor one copy each of fully-glycosylated and non-glycosylated PrP^C^. Right: Non-glycosylated PrP^C^ carrying N to glutamine (Q) substitution at residues 181 (N181Q) and 197 (N197Q). **b**, **c**: p^WM^-CJD brain homogenates (BH) were injected to TgHuPrP^Gly+/+^ (lanes 1-3) and to TgHuPrP^Gly+/-^ (lanes 4-6) mice. Mouse brains were homogenized with 1X DPBS to generate a 20% (wt/vol) BH, which were diluted (1:1) with 2X LB100 (pH 8.0). **b**: Unlike TgHuPrP^Gly+/+^, the unglycosylated PrP (*) is predominant in TgHuPrP^Gly+/-^ (mean±SD: 11.8±3.1 vs. 3.8±0.2; P<0.05). A smaller PrP fragment of ~ 19 kDa (**) is detected in TgHuPrP^Gly+/-^. Di (D)-, Mono (M)-, and Un (U)-glycosylated PrP isoforms. **c**: A simpler PrP profile is visualized following immunoblotting with tohoku-2. The ~19 kDa PrP fragment (**) is seen only in TgHuPrP^Gly+/-^. **d**: Merging of 3F4 and tohoku-2 channels (LI-COR). PrP bands were resolved using precast 12% Tris-Glycine (TGX), 8.7 cm-long gels.

**Fig. S3** Transgenic *PrP* sequences surrounding codons 181 and 197 in Tg mice carrying wild type or mutated human PrP transgenes. Mutated nucleotides and amino acid residues are indicated in red. **a**: PrP transgenes in the Tg(HuPrP^Gly+/+^) mice carry only asparagine (Asn, N) codon at both codons 181 and 197 (181NN/197NN). **b**: PrP transgenes in the Tg(HuPrP^Gly-/-^) mice carry only the mutated glutamine (Gln, Q) codon at both codons 181 and 197 (181QQ/197QQ). **c**: PrP transgenes in the heterozygous Tg(HuPrP^Gly+/-^) mice carry a wild-type human PrP^129M^ allele and a mutated human PrP^181Q/197Q^ allele. Overlapping nucleotide peaks are indicated with an asterisk (*).

**Fig. S4** Primary cleavage sites and proteinase K (PK)-resistant PrP^D^ (resPrP^D^) types of CJD. **a**: Schematic representation of full-length (i) and truncated (ii-iv) PrP^D^ species. Representation of primary (large arrows) and secondary (small arrows) PK cleavage sites, PK-sensitive and PK-resistant regions of PrP^D^. Primary and secondary PK cleavage sites for T1^21^ and T2 are indicated by red and green arrows, respectively. The unknown PK cleavage site for T1^20^ is indicated by the blue arrow. N-linked glycan and the disulfide bond have been omitted for clarity. N and C: N- and C- termini; G (glycine), S (serine), W (tryptophan). **b**: Western blot representation of the resPrP^D^ T1^21^ and T1^20^ variants and T2 of p-CJD. Numbers atop each resPrP^D^ band indicate the relative molecular weight.

**Fig. S5** Western blot profile of PrP harvested from the cerebellar white matter in p^WM^-CJD, sCJDMM1 and -MM2. **a**-**c**: White matter was homogenized with LB100 pH 8.0, and PrP extracted using a methanol/chloroform/water precipitation protocol; proteolytic digestion (**c**) was performed with 5 U/ml PK at 37 °C. **a** and **b**: WB profiles of total PrP in p^WM^-CJD and sCJD; **b**(i) and (ii) are different times of signal detection. **c**: PK digested white matter PrP was incubated with PNGaseF. Under our experimental conditions, resPrP^D^ is only partially diglycosylated. T1 unglycosylated resPrP^D^ isoform of p^WM^-CJD and sCJDMM1 migrates to ~ 20 kDa, whereas resPrP^D^ associated with sCJDMM2 migrates to ~ 19 kDa (T2). **c**(i) and (ii) are different times of signal detection. Each lane corresponds to a different case.

**Fig. S6** PK-titration assay of p^WM^-CJD. **a**: PK_1/2_, the amount of PK required to digest 50% of PrP^D^, is ~ 4-fold greater in T1^21^ than T1^20^ (60±10 vs. 14±3 U/ml; P<0.005). **b**: Representative WB of T1^21^ (top) and T1^20^ (bottom) variants. Numbers atop each unglycosylated resPrP^D^ band indicate the relative molecular weight.

**Fig. S7** Periodic acid–Schiff staining. **a**: A white matter (WM) kuru plaque. **b**: A kuru plaque affecting the granular layer (Grl. L.); Mol. L: molecular layer.

**Fig. S8** Aβ and tau pathologies. **a**-**c**: p^GM^-CJD case 3; **d** and **e**: p^WM^-CJD case 19. **a**: Dense core Aβ plaque. **b**: Hematoxylin-eosin staining showing a dense core Aβ plaque; inset: anti-PrP PrP antibody labels the corona, but not the core of an Aβ plaque. **c**: Thioflavin S (ThS)-positive Aβ plaques; inset: ThS-positive cerebral amyloid angiopathy. **d**: Neurofibrillary tangles and dystrophic neurites in hippocampus CA1. **e**: Sub-ependymal tau pathology with torn-shaped astrocytes in the temporal horn of the lateral ventricle. Antibodies: 4G8 to Aβ (**a**), 3F4 to PrP (**b**, inset), AT8 to hyperphosphorylated tau (**d** and **e**).

**Fig. S9** Histopathology of partially glycosylated Tg mice inoculated with p^WM^-CJD. Hematoxylin-eosin staining (**a**-**d**) and PrP immunohistochemistry (**e** and **f**). **a**-**c**: Clustered unicentric plaques (arrows in **b** and **c**); plaques in **c** are in sub-ependymal position. **d**: Heavily clustered plaques with amorphous appearance (arrows). **e**: PrP staining of clustered plaques shown in **a**. **f**: PrP plaque in layer VI of the cerebral cortex (CC). WM: White matter; antibody: 3F4.

**Fig. S10** Schematic representation of resPrP^D^ gel mobility at different buffer pHs. At buffer pH 6.9, the unglycosylated (unglyc) isoform of resPrP^D^ T1 migrates as a major band of ~ 21 kDa (black rectangle) and an underrepresented fragment of ~ 20 kDa (green dashed line) in the cerebral cortex (CC) and subcortical regions (Subc) of sCJDMM1 and p-CJD ([4]; this study). At buffer pH 8.0, T1 appears as either a major fragment or the only fragment of ~ 20 kDa (green rectangle); a slower migrating faint band of ~ 21 kDa (black dashed line) can be detected when long gels are employed ([1–3]; this study). In iCJD with 129MM genotype (iCJD-129MM) and kuru plaques (KP), unglyc resPrP^D^ T1 migrates as a major fragment of ~ 20 kDa at pH 6.9 and 8.0. The dashed lines refer to underrepresented resPrP^D^ bands that may not be detected if stringent experimental conditions (adequate PK/Tris concentration and buffer pH) and more sophisticated equipment (near infrared vs. chemiluminescence) are not employed. Dotted line refers to an expected minor fragment of ~ 20 kDa when the above experimental conditions are met. #([6, 13]; this study), *([3, 5, 8, 10, 12]), **([4]), ^([5]).

**FIGURES**

**Fig. S1**


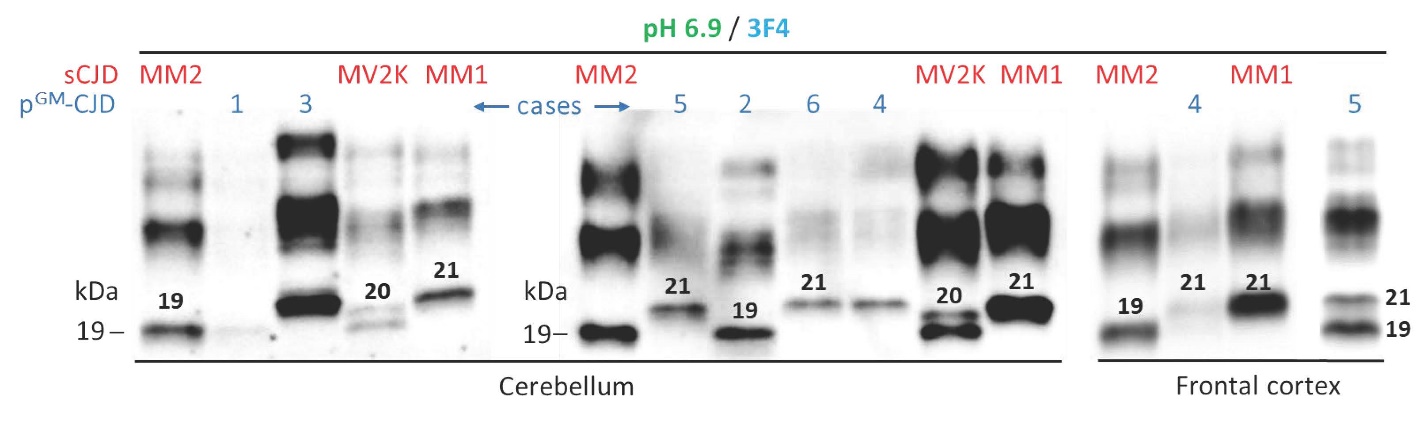


**Fig. S2**


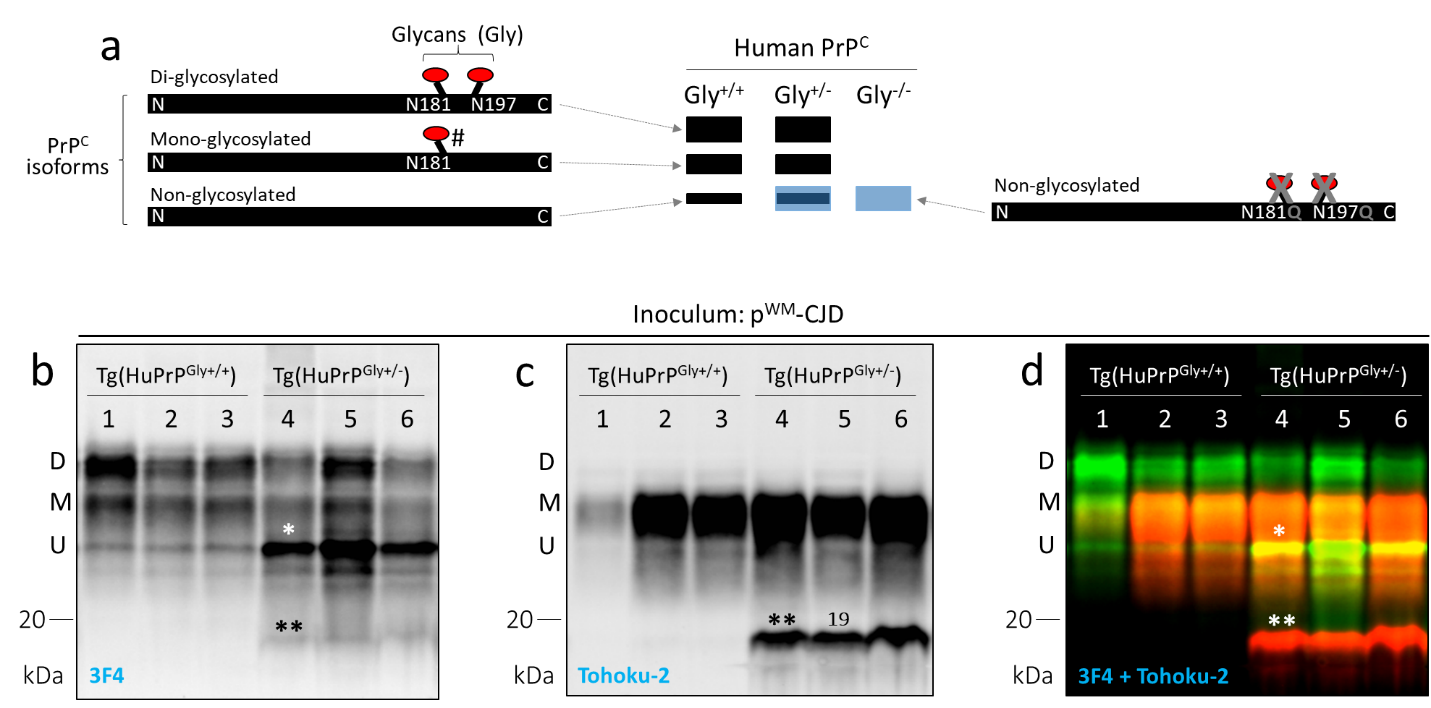


**Fig. S3**

**
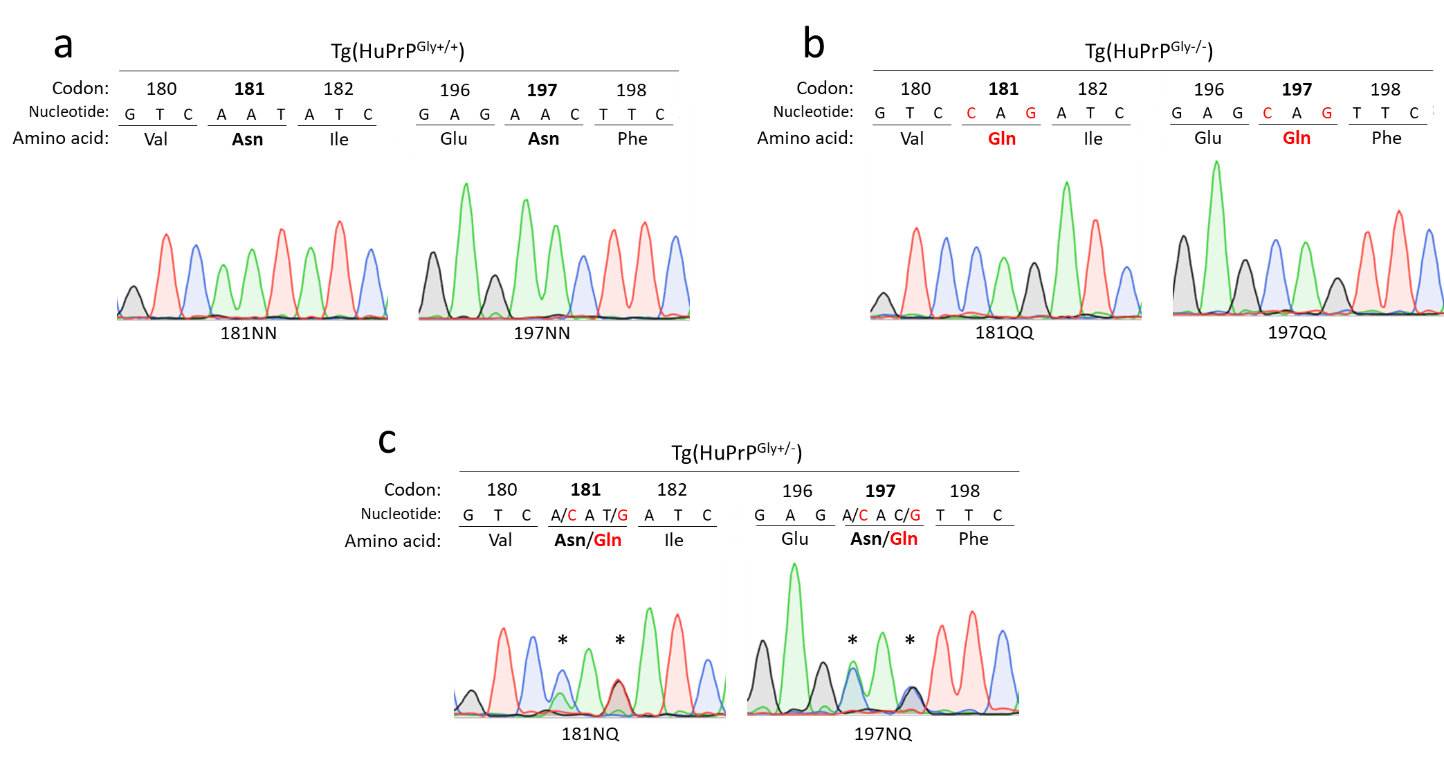
**

**Fig. S4**


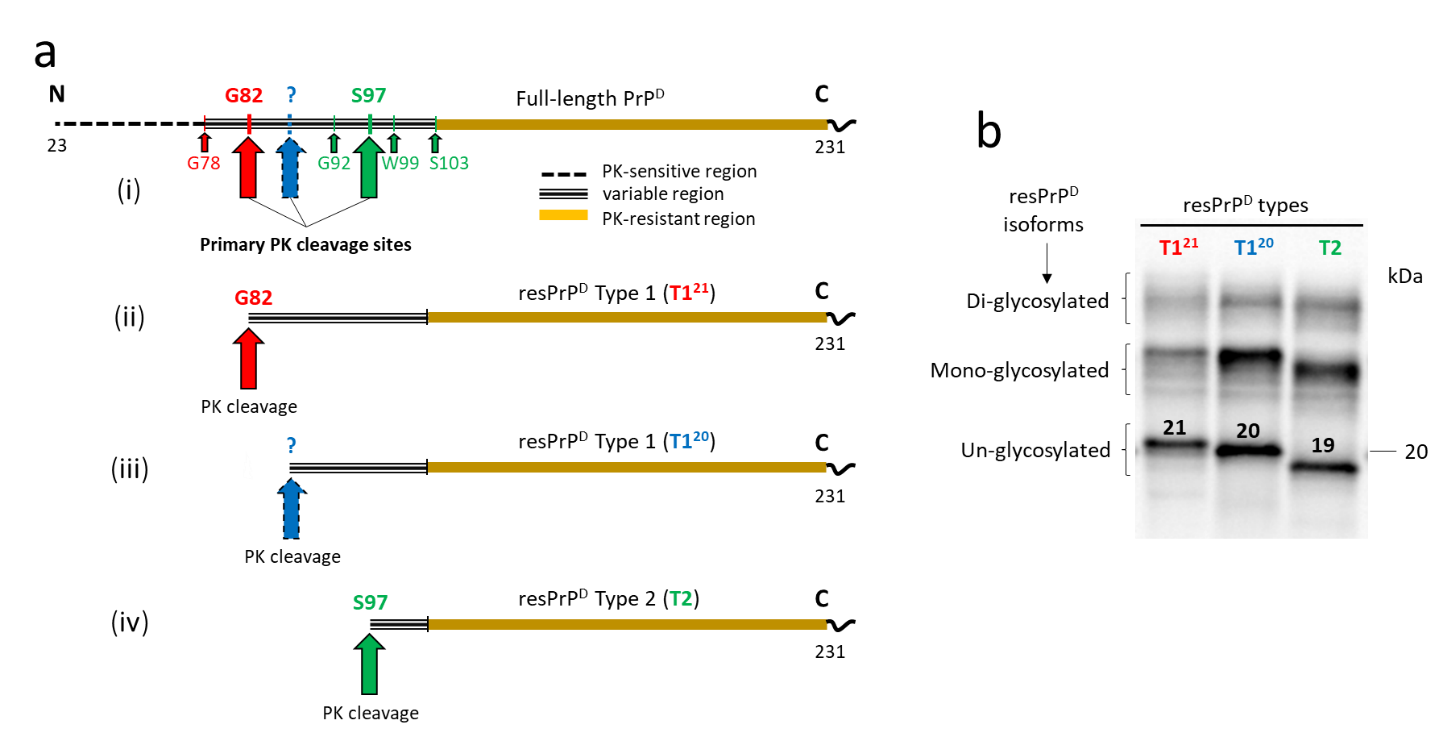


**Fig. S5**


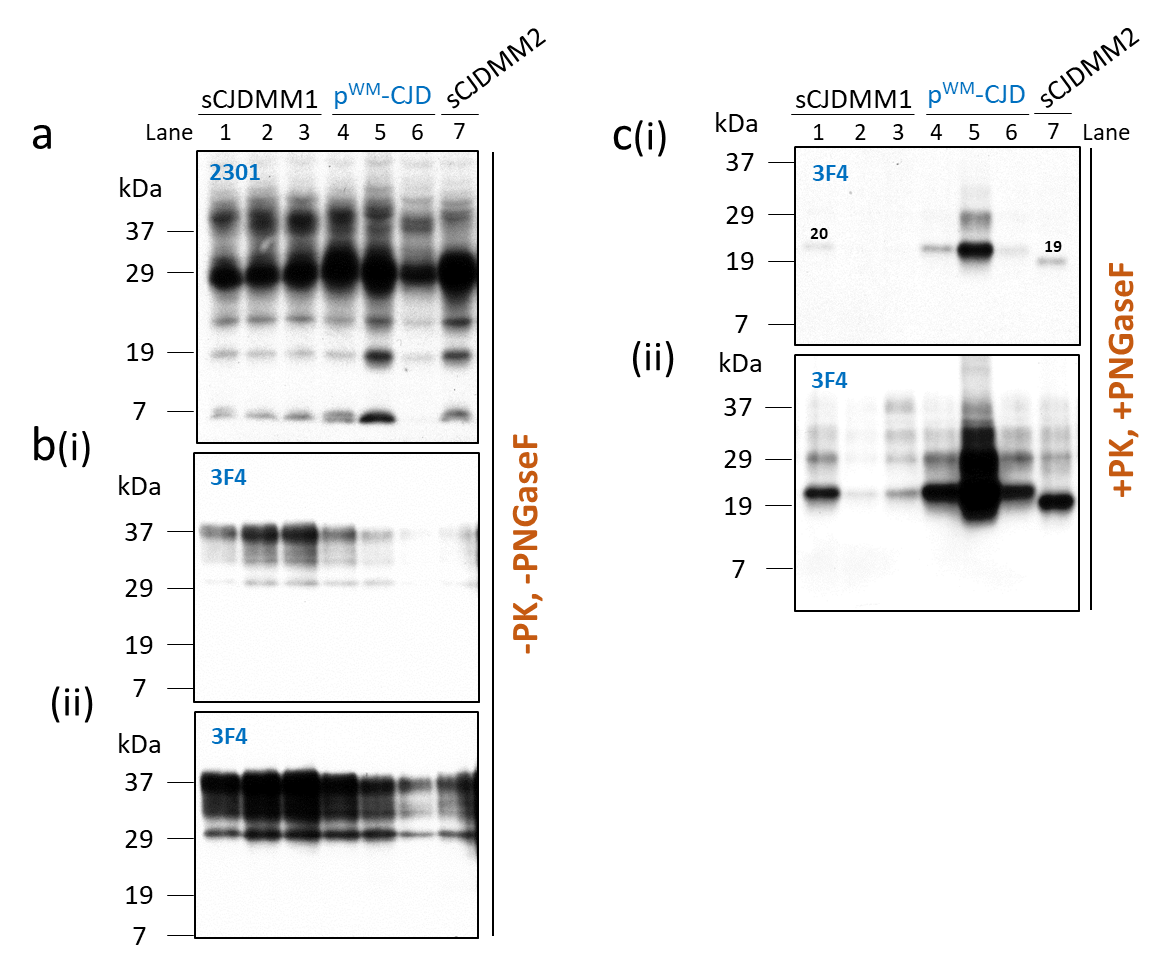


**Fig. S6**


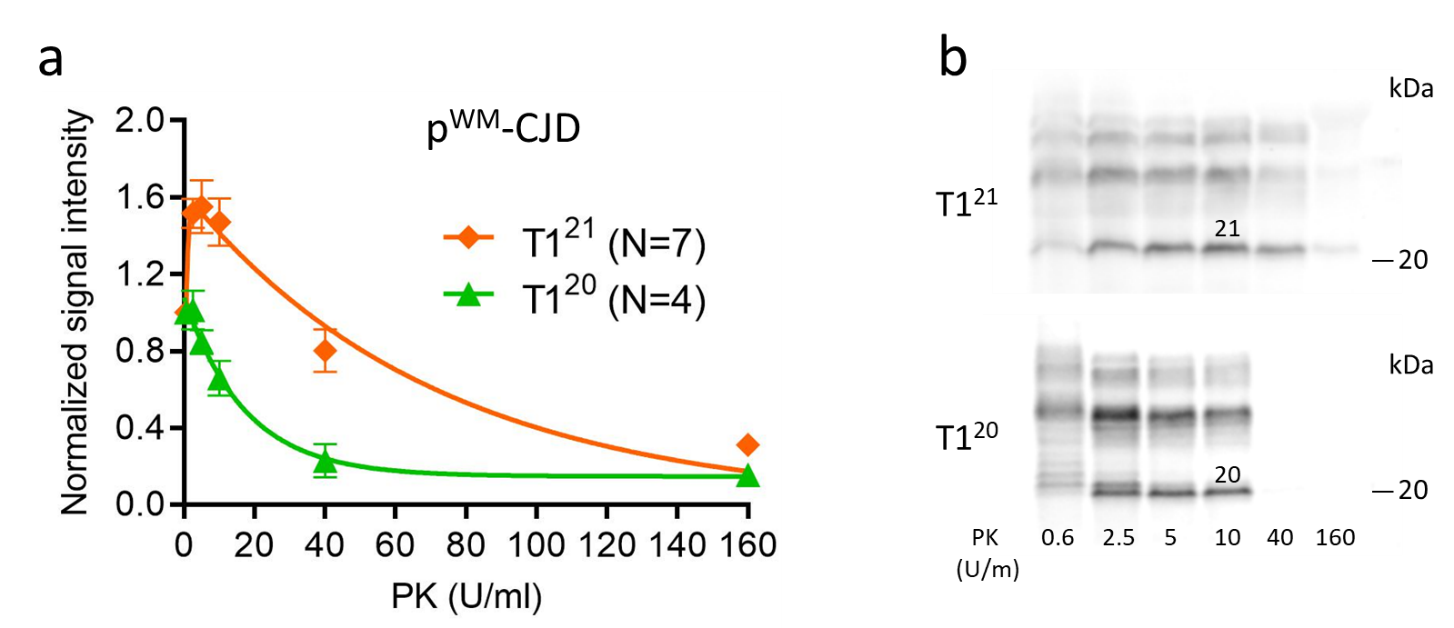


**Fig. S7**


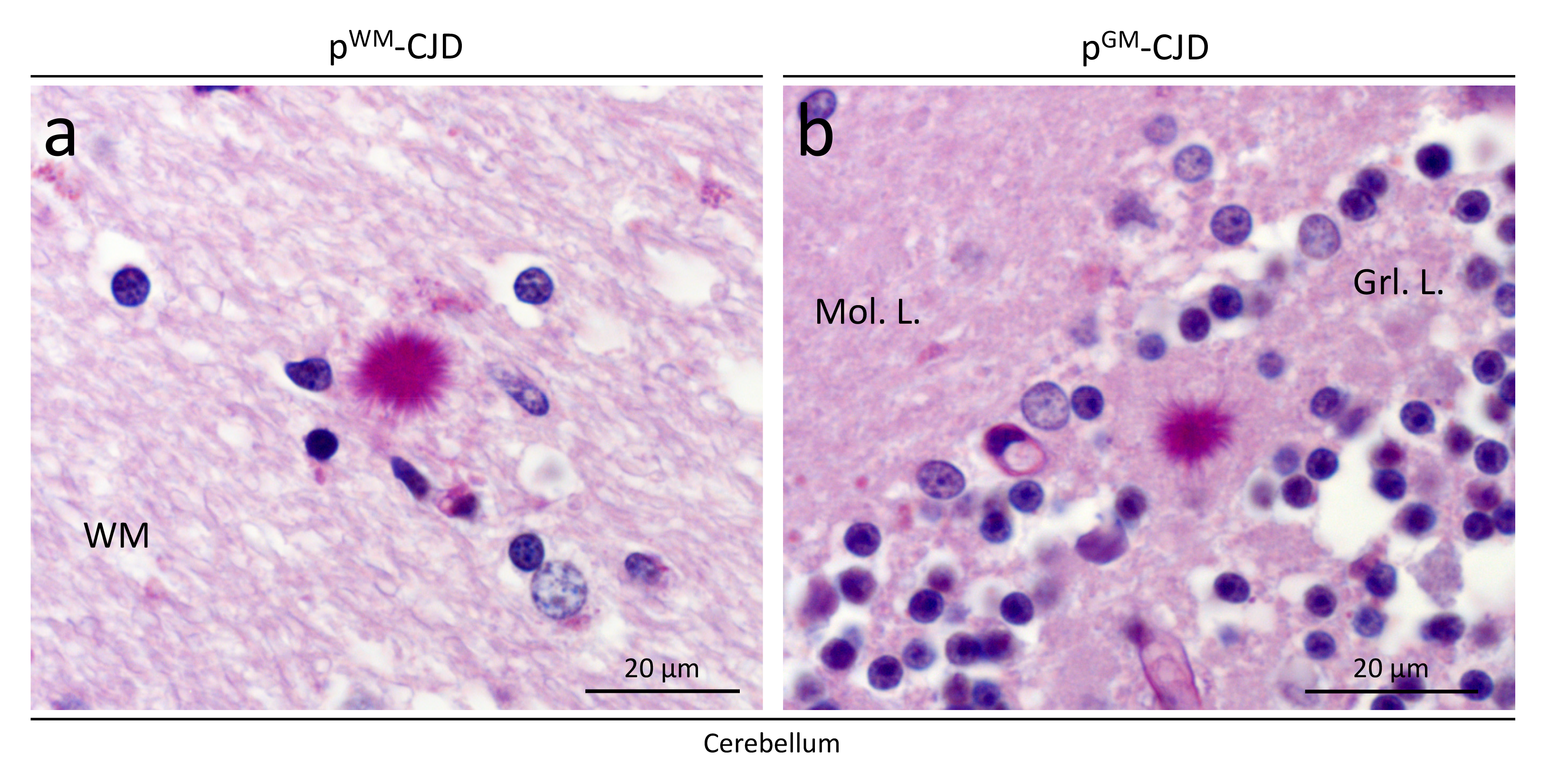


**Fig. S8**


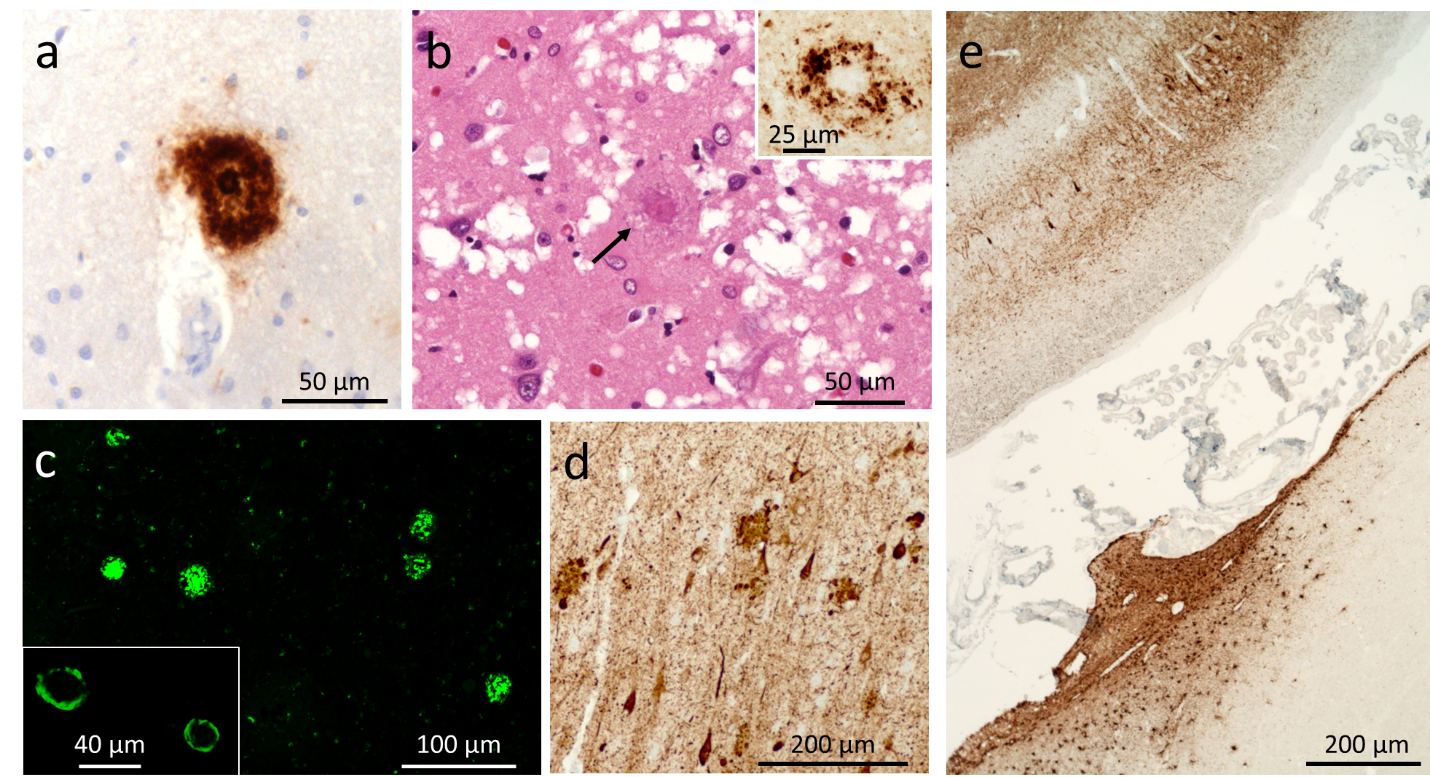


**Fig. S9**


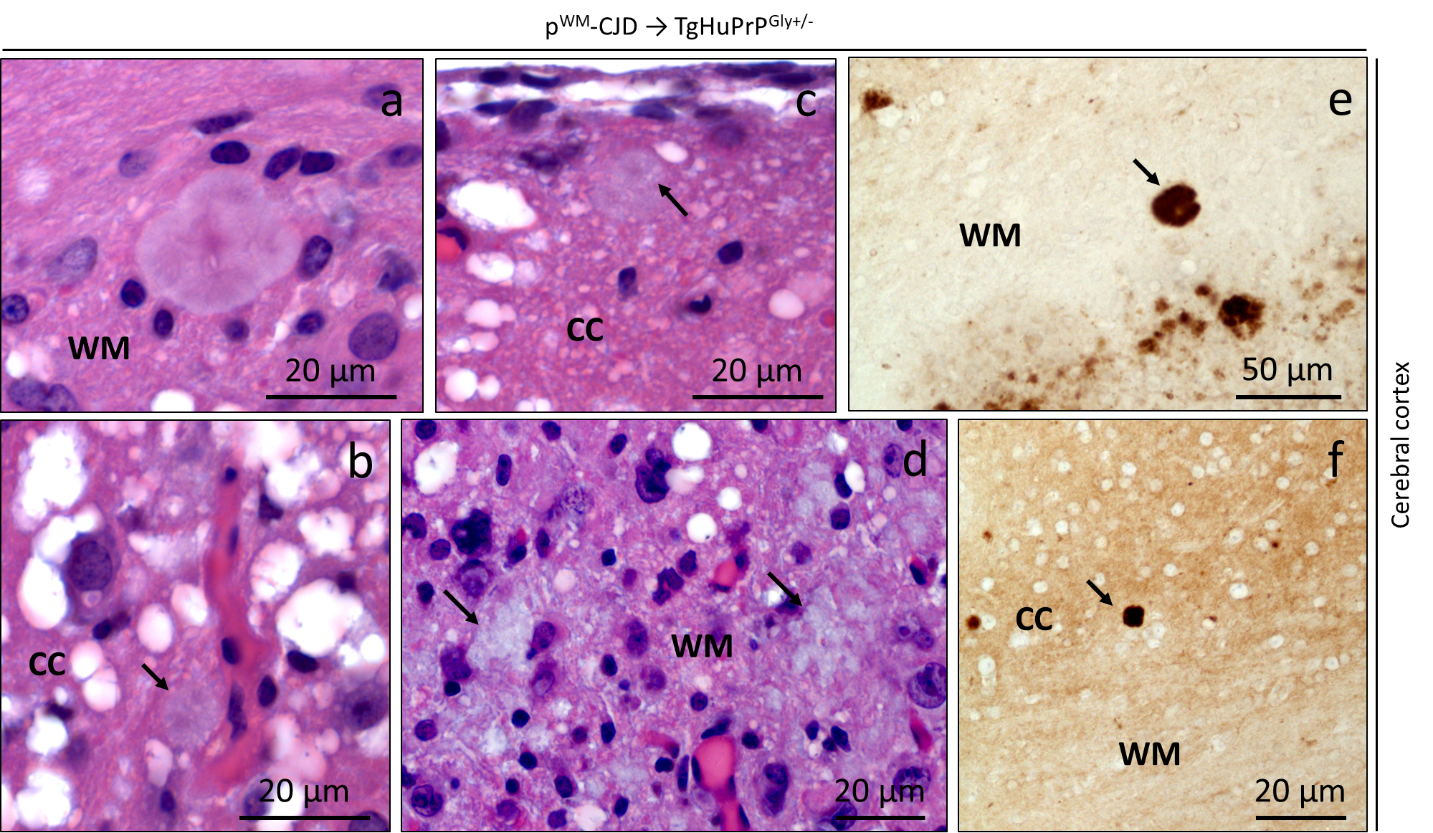


**Fig. S10**


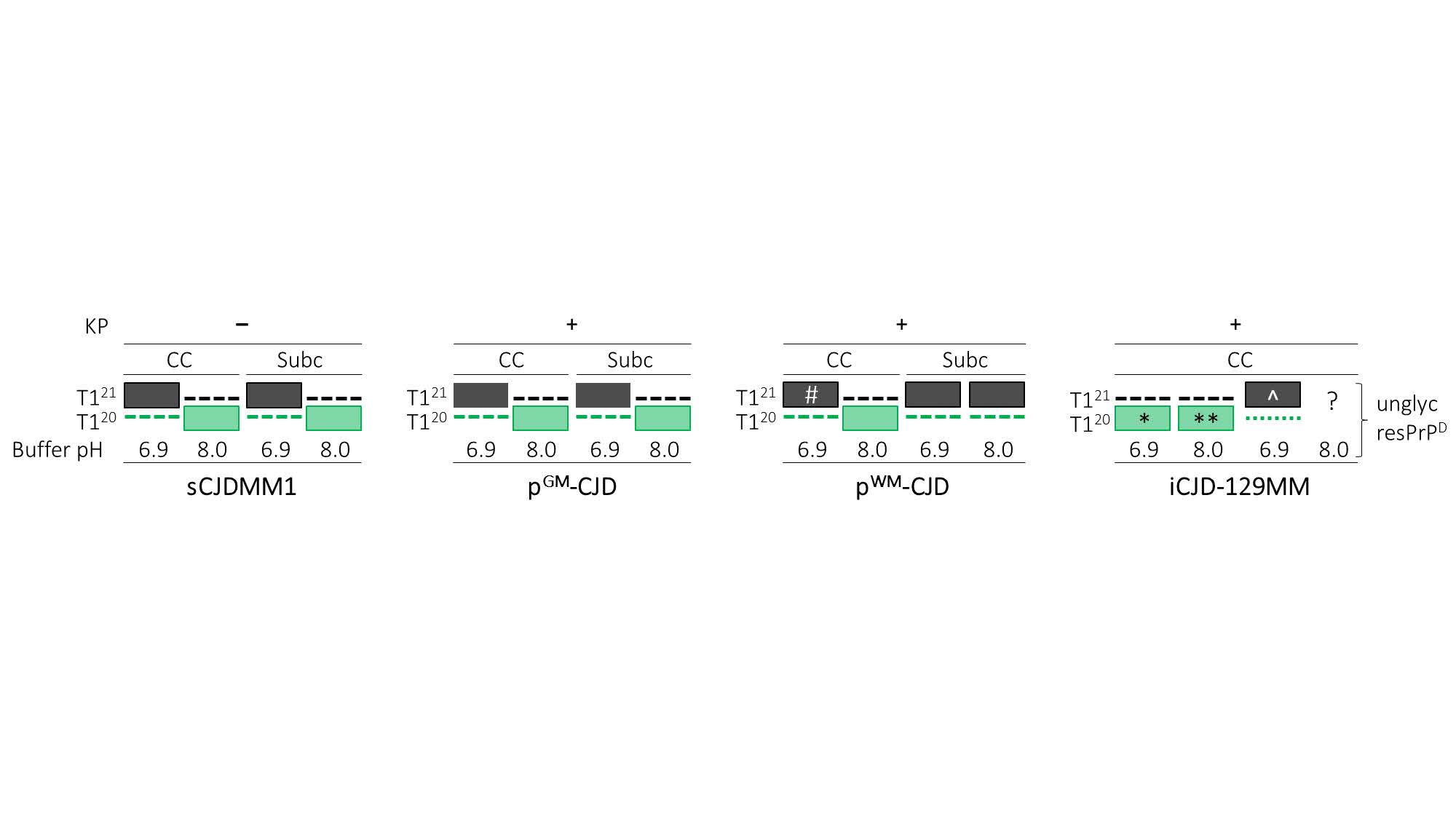

Supplement: Supplementary file 1 — Supplementary file1 (DOCX 17663 KB) [file 401_2023_2581_MOESM1_ESM.docx]
