# Supplementary material for: A novel subtype of sporadic Creutzfeldt–Jakob disease with PRNP codon 129MM genotype and PrP plaques
Source: Acta Neuropathol. 2023 May 8;146(1):121–43. doi: 10.1007/s00401-023-02581-1 (PMC10166463; doi:10.1007/s00401-023-02581-1)
Supplement: Supplementary file 2 — Supplementary file2 (DOCX 26 KB) [file 401_2023_2581_MOESM2_ESM.docx]

**Table S1** Prevalence of Aβ and tau pathologies in US p-CJD

| Prion disease | Aβ | | | | |  | | Tau | | |
| --- | --- | --- | --- | --- | --- | --- | --- | --- | --- | --- |
|  | Positive  cases ^a^ | CP | CAA + CP | CAA | Subpial |  | Positive cases ^a^ | | NFT | DN |
| p^GM^-CJD (n=7) | 86 ^b^  (6/7) ^c^ | 50  (3/6) | 50  (1/2) | 50  (1/2) | 17  (1/6) |  | 100  (7/7) | | 100  (7/7) | 14  (1/7) |
| p^WM^-CJD (n=14) | 50  (7/14) | 43  (3/7) | 33  (1/3) | 67  (2/3) | 43  (3/7) |  | 86  (12/14) | | 86  (12/14) | 7  (1/14) |

^a^ Assessed in frontal, temporal, occipital, hippocampal and entorhinal cortices; cases were considered positive when one or more brain regions showed Aβ or tau positive staining. ^b^ Percentage of cases with Aβ pathology. ^c^ Cases with positive staining/total cases examined. CP: Core plaque; CAA: cerebral amyloid angiopathy; NFT: neurofibrillary tangles; DN: dystrophic neurites. No differences found when comparing each variable (Chi-square and Fisher’s exact tests).
