# Supplementary material for: A novel subtype of sporadic Creutzfeldt–Jakob disease with PRNP codon 129MM genotype and PrP plaques
Source: Acta Neuropathol. 2023 May 8;146(1):121–43. doi: 10.1007/s00401-023-02581-1 (PMC10166463; doi:10.1007/s00401-023-02581-1)
Supplement: Supplementary file 3 — Supplementary file3 (DOCX 31 KB) [file 401_2023_2581_MOESM3_ESM.docx]

**Table S2** Presenting symptoms and risk factors of iatrogenic prion disease of US p-CJD subtypes

| Case number | Hunter/  (type) | Venison consumption | CWD  state ^a^ | Blood  transfusion | Travel to BSE-exposed countries ^b^ | Had acquired prion disease risk factor | Surgeries (n) | Surgeries description |
| --- | --- | --- | --- | --- | --- | --- | --- | --- |
| 1 | + (U) | + | + | + | – | – | + (1) | cardiac bypass (1992) |
| 2 | U | U | U | U | U | – | + (1) | basal cell carcinoma resection (2014) |
| 3 | – | – | U | – | – | – | U | U |
| 4 | – | – | U | – | + | – | + (3) | cataract (2016), two hip surgeries (2013) |
| 5 | – | – | U | – | – | – | – | U |
| 6 | – | – | U | – | – | – | + (4) | TAH-SO, knee, wrist surgery, knee surgery, cardiac cath. |
| 7 | – | – | U | – | + | – | – | U |
| 8 | – | – | U | – | – | – | U | U |
| 9 | – | – | U | U | – | U | U | U |
| 10 | – | – | U | – | – | – | + (1) | BPH surgery |
| 11 | U | U | U | U | U | + | + (4-6) | Multiple shunt revisions (77’-80’); pineal tumor surgery (79’) |
| 12 | – | – | U | U | – | – | – | – |
| 13 | + (D) | + | U | – | – | – | + (2) | Knee surgery, inguinal hernia |
| 14 | + (D) | + | + | – | – | – | – | U |
| 15 | + (D,E,M,C) | + | + | – | – | U | – | U |
| 16 | – | – | U | + | + | – | – | U |
| 17 | – | – | U | – | – | U | U | U |
| 18 | – | – | U | – | – | – | U | U |
| 19 | U | U | U | U | U | – | – | U |
| 20 | U | U | U | U | U | U | + (1) | Right nephrectomy |
| 21 | – | – | U | + | – | U | + (1) | Foot bone graft |

^a^ Consumed venison or hunted cervids in a CWD-endemic state as of January 2023; ^b^ during the period 1980-1996. None of the p-CJD cases have history of medical procedures with cadaveric growth hormone or dura mater. D: Deer; E: Elk; M: Moose; C: Caribou. TAH: Total abdominal hysterectomy; SO: Salpingo-Oophorectomy; BPH: benign prostatic hyperplasia; cath.: catheterization. U: Unknown.
