# Supplementary material for: A novel subtype of sporadic Creutzfeldt–Jakob disease with PRNP codon 129MM genotype and PrP plaques
Source: Acta Neuropathol. 2023 May 8;146(1):121–43. doi: 10.1007/s00401-023-02581-1 (PMC10166463; doi:10.1007/s00401-023-02581-1)
Supplement: Supplementary file 4 — Supplementary file4 (DOCX 25 KB) [file 401_2023_2581_MOESM4_ESM.docx]

**Table S3** Risk factors of iatrogenic prion disease in US p-CJD and age- and-gender-matched US sCJD cases

| Prion disease | Hunters % | Venison consumption | Blood transfusion | Travel in BSE affected country (1980-1996) | Recognized acquired prion disease risk factors | History of surgery |
| --- | --- | --- | --- | --- | --- | --- |
| p-CJD | 23  (4/17) ^a^ | 24  (4/17) | 20  (3/15) | 18  (3/17) | 6  (1/16) ^b^ | 56  (9/16) |
| sCJD | 25  (5/20) | 30  (6/20) | 5  (1/19) | 16  (3/19) | 0  (0/21) | 62  (10/16) |

^a^ Cases with the feature listed/total cases examined; ^b^ one patient (case 11) had several neurosurgeries related to VP shunts. No differences found when comparing each variable (Chi-square and Fisher’s exact test).
