# Supplementary material for: A novel subtype of sporadic Creutzfeldt–Jakob disease with PRNP codon 129MM genotype and PrP plaques
Source: Acta Neuropathol. 2023 May 8;146(1):121–43. doi: 10.1007/s00401-023-02581-1 (PMC10166463; doi:10.1007/s00401-023-02581-1)
Supplement: Supplementary file 5 — Supplementary file5 (DOCX 26 KB) [file 401_2023_2581_MOESM5_ESM.docx]

| Prion disease | Inoculum | |  | Tg(HuPrP^Gly+/+^) | | | |  | Tg(HuPrP^Gly+/-^) | | | |
| --- | --- | --- | --- | --- | --- | --- | --- | --- | --- | --- | --- | --- |
|  | Brain region | resPrP^D^ |  | Dpi | resPrP^D^ | N mice | PrP plaques |  | Dpi | resPrP^D^ | N mice | PrP plaques |
| p^WM^-CJD | Putamen | T1^21-20^-T2 ^a^ |  | 233±6 | T1^20^ | 2 | + |  | 235±37 | T2 | 5 | + |
| sCJDMM1 | Putamen | T1^20^ |  | 183±3 | T1^20^ | 2 | – |  | 272±38 | T1^20^ | 3 | na |

**Table S4** Transmission features of p^WM^-CJD and sCJDMM1 prions with fully glycosylated and partially glycosylated TgHuPrP mice

^a^ PK-resistant PrP^D^ (resPrP^D^) isoform with T1^21-20^ coexisting with T2 (T1^21-20^-T2); Tg(HuPrP^Gly+/+^): mice expressing wild-type, fully glycosylated human PrP; Tg(HuPrP^Gly+/-^): mice expressing a muted (asparagine, N, replaced by glutamine, Q) (PrP-N181Q/N197Q), partially glycosylated human PrP; dpi: days post-inoculation (expressed as mean±SD); na: not available.
